# Generalizable Strategies for the Synthesis of Cereblon‐Recruiting PROTAC Prodrugs

**DOI:** 10.1002/anie.3238473

**Published:** 2026-08-05

**Authors:** Aiden X. Wang, Bin Liu, Elise M. Ackerman, Yichen Xiang, Hung V.‐T. Nguyen, Yivan Jiang, Angela N. Koehler, Jeremiah A. Johnson

**Affiliations:** ^1^ Department of Chemistry Massachusetts Institute of Technology Cambridge Massachusetts USA; ^2^ Koch Institute for Integrative Cancer Research Massachusetts Institute of Technology Cambridge Massachusetts USA; ^3^ Broad Institute of MIT and Harvard Cambridge Massachusetts USA; ^4^ Department of Biological Engineering Massachusetts Institute of Technology Cambridge Massachusetts USA; ^5^ Center For Precision Cancer Medicine Massachusetts Institute of Technology Cambridge Massachusetts USA

**Keywords:** alkylation, cereblon, chemistry, combinatorial chemistry, cytotoxicity, linker, macromonomer, prodrug, protein degradation

## Abstract

Cereblon (CRBN)‐recruiting PROTACs (proteolysis‐targeting chimeras) are among the most clinically advanced degraders but remain challenging to chemically modify for translation to prodrug‐based delivery strategies. Here, we report three orthogonal approaches—triazole quaternization, tertiary‐amine alkylation, and installation of a hydroxyl linker—that enable chemoselective, high‐yielding syntheses of CRBN‐recruiting PROTAC prodrugs. These strategies allow incorporation of self‐immolative linkers whose cleavage kinetics can be predictably tuned to release the parent PROTAC, which is demonstrated in the context of PEGylated macromonomer and bottlebrush prodrug macromolecular scaffolds. In multiple myeloma models, representative PROTAC‐bottlebrush prodrugs (PROTAC‐BPDs) induce cellular potency profiles that follow the designed PROTAC release rates, confirming that the observed protein degradation and cytotoxicity arise from effective prodrug linker cleavage. Collectively, this work establishes generalizable approaches for constructing prodrugs of CRBN‐based PROTACs, expanding the synthetic space for targeted protein degradation and providing new design principles for controlling degrader activation, selectivity, and in vivo delivery.

## Graphical abstract

Cereblon‐recruiting PROTACs remain challenging to derivatize for prodrug‐based delivery. In this study, three complementary conjugation strategies enabled the efficient synthesis of cleavable PROTAC prodrugs with tunable release kinetics that can be incorporated into a library of PEGylated constructs and bottlebrush prodrugs. Representative examples displayed controlled degrader release, potent anticancer activity in vitro, tumor accumulation, and preliminary in vivo efficacy.

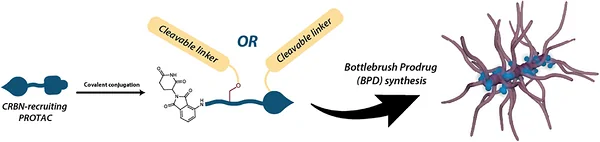

## 1 Introduction

Proteolysis targeting chimera (PROTAC)–induced protein degradation is an emerging therapeutic modality for cancer and other disease contexts [1, 2]. Typically, PROTACs take the form of heterobifunctional molecules comprising two ligands connected with a linker: one ligand binds a protein of interest (POI), while the other binds an E3 ligase. Upon simultaneous binding of both the POI and E3 ligase by the PROTAC, ubiquitination of the POI is induced, resulting in its eventual degradation via the ubiquitin‐proteasome system (UPS) [3, 4, 5]. Recent advancements have greatly expanded the scope of POIs PROTACs are capable of degrading [6, 7], and PROTACs continue to show promise in preclinical and clinical contexts [8]. Nevertheless, the scope of E3 ligases used for PROTAC design is relatively limited. Out of the over 600 E3 ligases in the human proteome, > 90% of PROTACs currently in clinical trials engage CRBN, while VHL accounts for majority of the remaining examples [9]. Accordingly, both are relied on heavily in early PROTAC development [10, 11, 12, 13, 14].

Despite rapid developments over the past decade, the translation of PROTACs faces hurdles often related to factors like poor in vivo pharmacokinetics, off‐target toxicity, and low cell permeability [15, 16, 17, 18]. The development of prodrug variants of PROTACs could address these challenges [19]. Inspired by classical small‐molecule prodrugs, PROTAC prodrugs incorporate cleavable linkers that liberate the active degrader in response to an innate biological trigger, such as hypoxia [20], glutathione [21], and enzyme activity [22, 23], or an externally applied stimulus such as light [24, 25] and induced bioorthogonal cleavage [26, 27]. These approaches have demonstrated improvements in the tumor selectivity [20, 21, 27] and off‐tissue toxicity of PROTACs [20, 21, 27].

Despite this progress, it is notable that a majority of PROTAC prodrugs are based on VHL‐recruiting PROTACs, which is perhaps unsurprising as the secondary hydroxyl group of the hydroxyproline component of VHL ligands offers a convenient handle for prodrug derivatization under mild conditions [26, 27, 28, 29, 30] (Figure 1a). This privileged functionality has enabled prodrug designs and further derivatization for delivery applications, but it also means that the large class of cereblon (CRBN)‐recruiting PROTACs, which lack simple functional handles yet currently dominate the clinical landscape, remain underrepresented in the PROTAC prodrug space. A few reports describe the syntheses of prodrugs of CRBN‐recruiting PROTACs leveraging the glutarimide functionality of the thalidomide family of CRBN ligands (Figure 1b). For example, Xue et al. [25] and Liu et al. [31] introduced 4,5‐dimethoxy‐2‐nitrobenzyl (DMNB) derivatives that release free PROTACs upon UV light activation; Chen et al. [32] installed disulfide linkages that cleave and release upon reduction by endogenous GSH; and Chen et al. [22] used peptides that cleave upon Cathepsin B activation. Ring‐opened glutarimides that leverage endogenous prodrug activation mechanisms of imide cyclization have also recently been described [33]. Alternatively, Xue et al. described the de novo synthesis of BRD4‐degrading CRBN PROTACs with cleavable linkers installed at the amide nitrogen of the POI‐binding component [25]. While these strategies are promising, they suffer from common limitations of low yield (often not exceeding 10%), lack direct applicability to commercially available PROTACs, and/or the need for de novo synthesis for each prodrug. As a result, generalizable strategies for the synthesis of CRBN‐PROTAC prodrugs, ideally from existing PROTACs or via linker modification, remain desirable.

**FIGURE 1:**
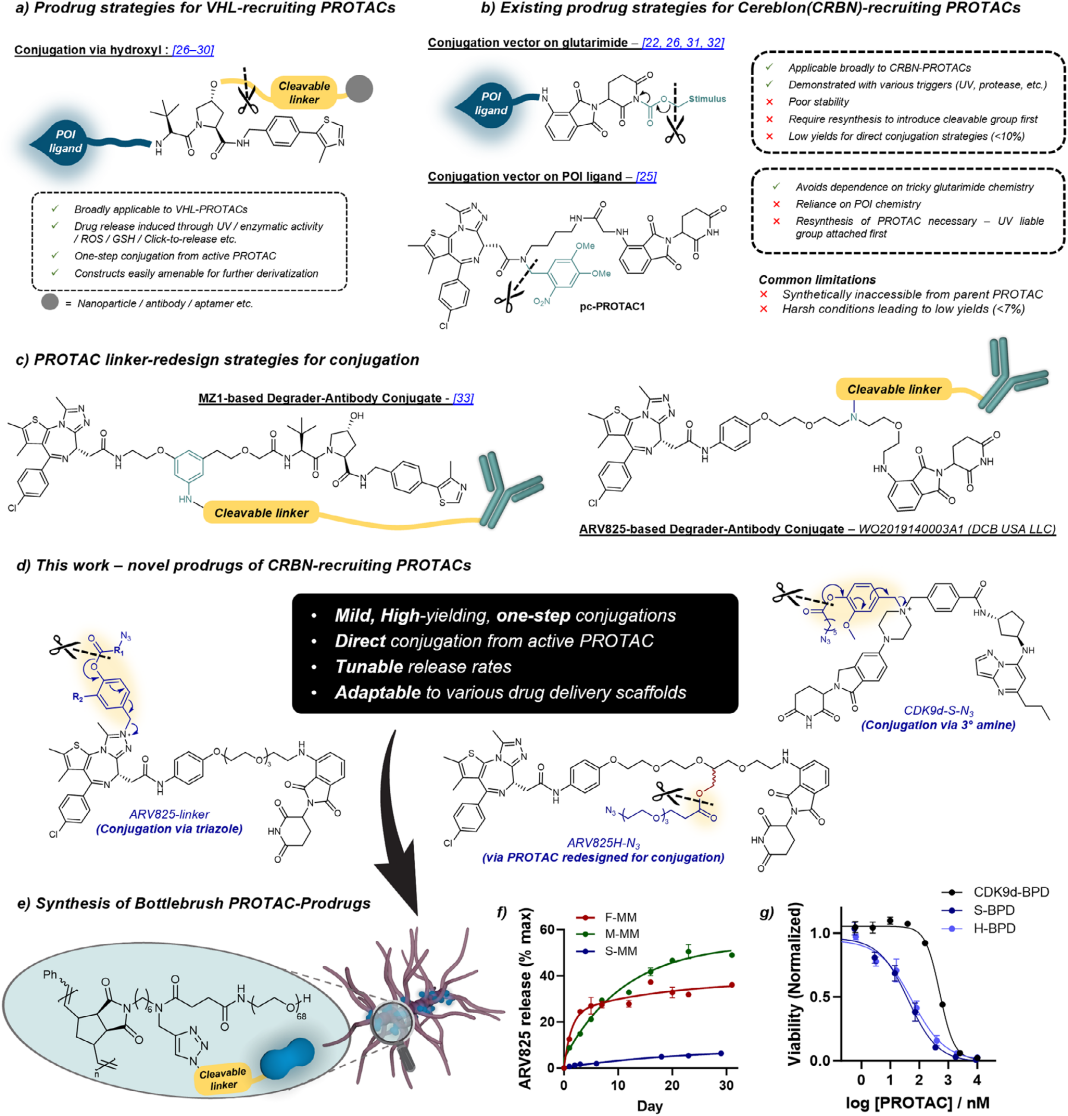
(a) Covalent conjugation can be readily performed on VHL‐based PROTACs through the hydroxyproline residue of the VHL‐binding ligand, enabling broad application of VHL‐based PROTACs as prodrugs and payloads for drug delivery scaffolds [23, 26, 27, 28, 29]. (b) Robust covalent conjugation strategies for cereblon (CRBN)‐based PROTACs are limited, citing common limitations of low yield and inaccessibility from the parent PROTAC [22, 26, 31, 32]. (c) Linker modification strategies enable conjugation to form degrader‐antibody conjugates [34]. (d) PROTAC prodrug synthesis through triazole or tertiary amine sites enables robust, high‐yielding attachment of self‐immolative linkers to CRBN‐based PROTACs, while redesigned PROTACs bearing an added –CH_2_OH handle provide an orthogonal route for conjugation. (e) Application of these conjugation strategies enables direct synthesis of PROTAC–bottlebrush prodrugs (PROTAC‐BPDs) from active degraders, demonstrating (f) welldefined release kinetics and (g) potent anti‐myeloma efficacy.

To address this gap, we set out to identify unconventional sites for prodrug design within CRBN‐PROTAC scaffolds. Rather than relying on conventional handles on the POI or CRBN‐binding regions (–OH, –NH_2_, –CO_2_H), we asked whether less commonly exploited motifs, such as triazoles and tertiary amines, could be leveraged for traceless PROTAC release. Here, we report syntheses of CRBN‐targeting PROTAC‐prodrugs (Figure 1d), that leverage such strategies in the context of two different CRBN‐PROTACs: ARV825 and KI‐CDK9d‐32. ARV825 is a BRD4‐targeting PROTAC that exhibits low‐nanomolar activity across several cancer cell lines and is frequently studied as a therapeutic modality in MYC‐driven malignancies such as multiple myeloma and neuroblastoma [35, 36, 37, 38]. KI‐CDK9d‐32 is a recently reported CDK9 degrader with sub‐nanomolar potency and rapid kinetics, leading to suppression of MYC expression and disruption of transcriptional programs [39]. These two scaffolds serve as distinct test cases to evaluate whether our derivatization strategies could extend across different CRBN‐recruiting architectures.

Structural biology studies [40, 41] often reveal a non‐binding role of PROTAC linkers, that is, where the linker adopts a solvent‐exposed position when bound to CRBN and POIs. This observation suggests that modest substitutions along the linker, such as a small functional handle to enable conjugation, could be tolerated without disrupting ternary complex formation. Indeed, such linker modification represents another strategy for PROTAC prodrug synthesis, with examples including incorporation of an aniline into the linker of MZ1 [34] and the replacement of an oxygen atom by nitrogen in the ARV825 linker (WO2019140003A1); both approaches permitted conjugation of the PROTAC to an antibody via a protease‐cleavable linker (Figure 1c). Guided by these observations, we designed ARV825H‐N_3_ to explore the effects of small modifications to the PROTAC linker as a complementary approach to triazole and amine quaternization for PROTAC prodrug design (Figure 1d).

Enabled by these strategies, we take a further step and demonstrate the synthesis of bottlebrush prodrugs (BPDs) that display controlled PROTAC release kinetics and exhibit potent anticancer efficacy against multiple myeloma cells (Figure 1e–g). Altogether, this work establishes new strategies for CRBN‐PROTAC prodrug design and provides a general framework for expanding degrader modalities into promising delivery platforms.

## 2 Results and Discussion

### 2.1 Synthesis of ARV825 Prodrugs via Triazole Benzylation

While conjugation strategies for CRBN‐based PROTACs have largely focused on derivatizing the glutarimide ring, the synthetic constraints of modifying this site prompted us to explore alternative approaches. Heterocycles based on the 1,2,4‐triazole motif have become increasingly prevalent in drug design; they are featured in numerous approved drugs [42] and PROTACs such as dBET6, QCA570, and ARV825. This growing presence across biologically active scaffolds represents a valuable foundation for new conjugation strategies.

Inspired by precedents in the context of small‐molecule BRD4 inhibitors [43], we identified triazole quaternization with benzylic electrophiles as a promising strategy to install traceless, cleavable prodrug linkers. Using ARV825 as a model substrate (Figure 2a), a series of phenyl ester‐based benzylic electrophiles with varying reactivity were investigated (Figure 2b); prodrug activation in such systems proceeds through cleavage of the *para*‐aryl ester followed by 1,6‐elimination to release ARV825 (Figure 1c). An azide terminus was included to enable later conjugation of these prodrugs to scaffolds of interest, for example, nanoparticles [29] or biomolecules [28]. A “slow” releasing linker (S), as determined by the linker hydrophobicity and aryl substituents [44], was used for initial reaction condition screening (Figure 2c, Entry 2; Figure S1). Ultimately, we found that heating S and ARV825 in a sealed microwave vessel at 120 °C for 6 h in acetonitrile gave the highest isolated yield (79%) of the quaternized ARV825 azide prodrug “ARV825‐S‐N_3_” following purification via neutral alumina column chromatography (Figure 2c, Entry 3). Notably, these products were obtained without requiring rigorously anhydrous conditions or HPLC purification. ^1^H NMR spectroscopic analysis of the crude S reaction mixture at *t* = 0 compared to the purified ARV825‐S‐N_3_ revealed distinct downfield shifts of proton resonances (e.g., labeled 1–6 in Figure 2d), consistent with successful conjugation (Figure S1). ^1^H‐^13^C HMBC correlations support the regioselectivity of conjugation, indicating that benzylation proceeds selectively at the less hindered triazole nitrogen (Figures 2e and S2). Comparable results were obtained under similar conditions using ester linkers that hydrolyze at “medium” (M) and “fast” (F) rates relative to S (Entries 6 and 7), providing prodrugs ARV825‐M‐N_3_ and ARV825‐F‐N_3_. Collectively, these results suggest that triazolodiazepine quaternization of ARV825 is selective under simple reaction conditions and compatible with the intact glutarimide moiety. This strategy thus provides a practical framework for extending conjugation and prodrug linker design to other CRBN‐based PROTACs containing triazole motifs.

**FIGURE 2:**
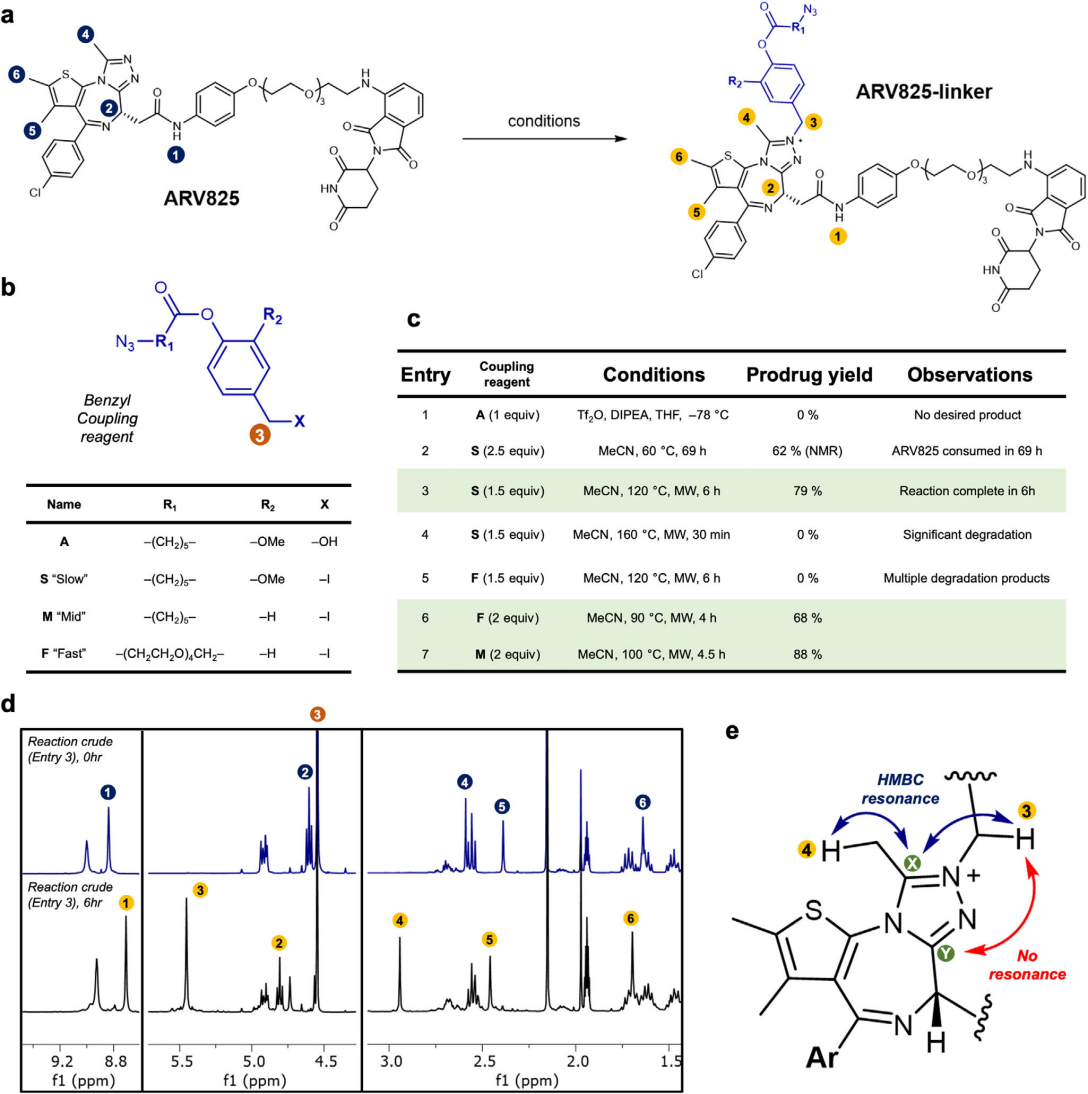
(a) Synthesis of ARV825‐S‐N_3_ through triazole‐mediated conjugation directly from the parent PROTAC ARV825. (b) Structures of benzyl coupling reagents evaluated for conjugation, differing in substituents and leaving groups. (c) Optimization of reaction conditions and coupling reagents for triazole conjugation. Highlighted entries indicate optimized conditions. (d) ^1^H NMR comparison of reaction crude (Entry 3) at *t* = 0 and ARV825‐S‐N_3_ showing distinct downfield shifts of signals near the conjugation site. Minor residual peaks in the purified conjugate spectrum arise from trace ARV825 release during workup or dissolution in the NMR solvent. (e) HMBC correlations confirm linkage exclusively through the less hindered triazole nitrogen.

### 2.2 Synthesis of KI‐CDK9d‐32 Prodrugs via Tertiary Amine Benzylation

We next asked whether this benzylation strategy could be extended to PROTACs containing tertiary amine groups, as has been shown for small molecules [45, 46]. Tertiary amines are increasingly common in PROTACs, often appearing in the form of piperidine and piperazine heterocycles [47]. These functionalities can improve solubility and introduce conformational rigidity in the place of linear alkyl or oligoethylene glycol‐based linkers, as seen in clinically advanced degraders like ARV‐110 and ARV‐471. Motivated by this trend, we sought to evaluate whether the phenyl‐ester‐based benzylic linkers used above could be installed onto a piperazine‐containing, recently reported CDK9‐degrading PROTAC, KI‐CDK9d‐32 (abbreviated to CDK9d).

To this end, KI‐CDK9d‐32 was combined with S and subjected to N‐benzylation under similar conditions to those established above for triazole conjugation but using N,N‐dimethylformamide (DMF) as the solvent due to the limited solubility of KI‐CDK9d‐32 (Figure 3a). Gratifyingly, the desired prodrug CDK9d‐S‐N_3_ was obtained in 40% yield following HPLC purification. ^1^H NMR spectroscopy revealed downfield shifts of the benzyl and piperazine proton resonances 1–3 in the product relative to the starting PROTAC (Figure 3b). ^1^H NOESY and ^1^H–^1^ ^3^C HMBC spectra indicated selective benzylation of the trialkyl‐amine component of KI‐CDK9d‐32, consistent with it being the most nucleophilic site in KI‐CDK9d‐32 (Figures 3c and S3). These findings suggest that cleavable linkers can be installed onto tertiary‐amine‐containing PROTACs via this quaternization strategy.

**FIGURE 3:**
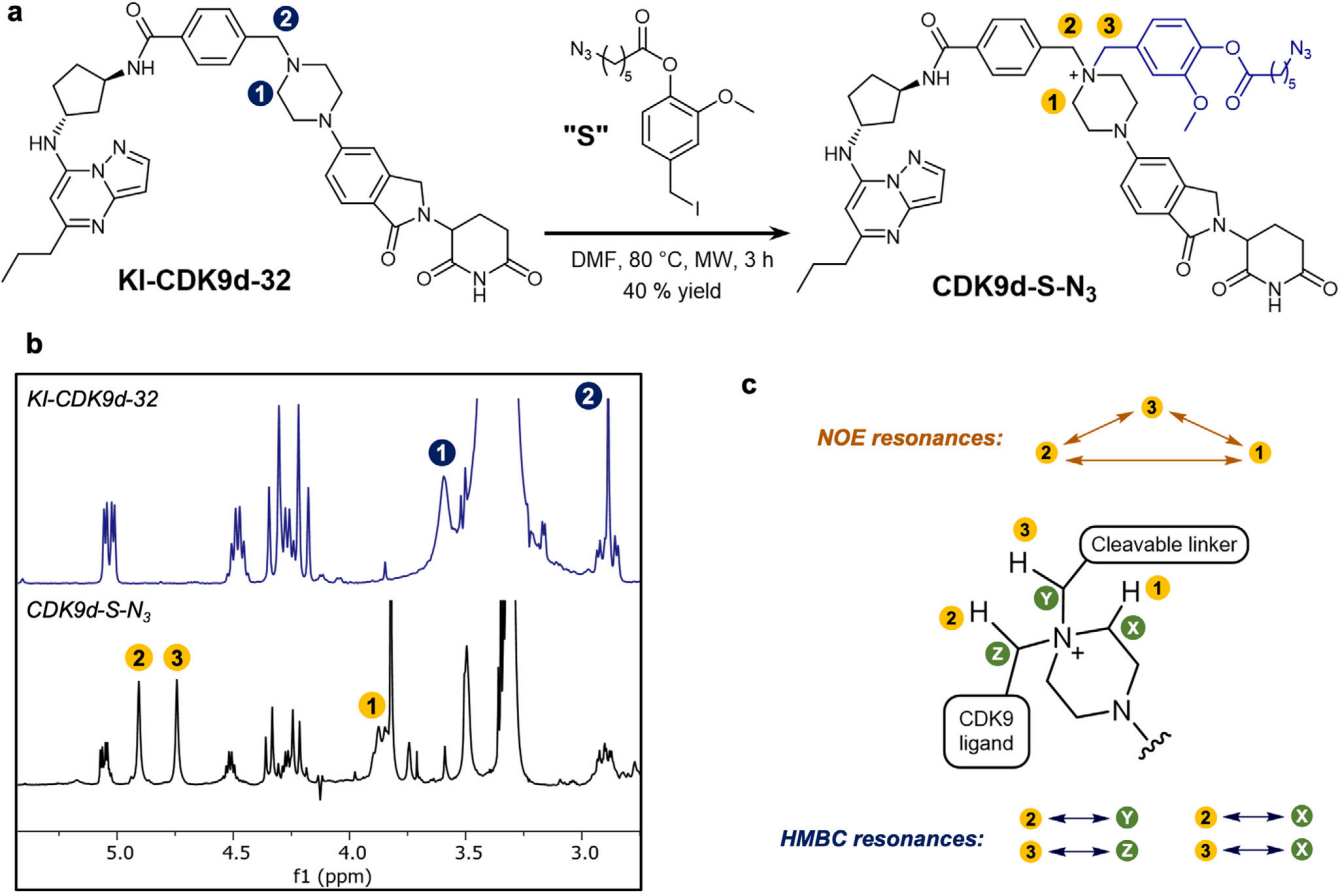
(a) Synthesis of CDK9d‐S‐N_3_ through tertiary amine‐mediated conjugation directly from the parent PROTAC KI‐CDK9d‐32. (b) ^1^H NMR comparison of KI‐CDK9d‐32 and purified CDK9d‐S‐N_3_ shows distinct downfield shifts of resonances near the conjugation site, validated by (c) NOE and HMBC correlations, which confirm linkage through the piperazine amine.

### 2.3 Design and Synthesis of ARV825H to Facilitate Conjugation

The above strategies are inherently limited to CRBN‐based PROTACs in which the POI‐binding ligand contains an accessible heterocyclic or tertiary amine‐containing functional group for derivatization. As noted above, ternary complex crystal structures of degraders like dBET6, dBET23, and CFT1297 (Figure S4) reveal binding poses where the linker region is exposed to solvent (water), suggesting that minimal modifications to the linker would be unlikely to disrupt ternary complex formation. To this end, we explored an analogous approach to prodrug design that targets the linker region as a universal site for PROTAC modification. Specifically, we designed and synthesized ARV825H, a derivative of ARV‐825 bearing an additional CH_2_OH group along the tetraethylene glycol (TEG) linker (Figure 4a). This handle was introduced through stepwise modification of the TEG linker segment: nucleophilic ring opening of **1** with 2‐azidoethanol, followed by chain extension using a tosylated diethylene glycol unit to afford **3**, onto which OTX015 and pomalidomide were sequentially conjugated. Subsequent THP deprotection of **5** under mild conditions yielded ARV825H as a mixture of diastereomers, which features the same linker length as ARV825 yet possesses a hydroxyl group for conjugation. ARV825H displayed similar ternary complex formation compared to its parent ARV825 in an AlphaScreen assay (Figure 4c). Moreover, complete BRD4 degradation was observed in multiple myeloma cells after 24 h exposure to ARV825H at concentrations as low as 40 nM, with re‐emergence of BRD4 at higher doses, behavior characteristic of the “hook effect” commonly observed for PROTACs (Figures 4b and S5) [48]. These results suggest that CH_2_OH addition to the TEG linker of ARV825 has minimal impact on its function. Additionally, a stereochemically pure variant of ARV825H synthesized from (+)‐glycidol exhibited comparable cytotoxicity to the mixture of diastereomers in KMS11 cells, indicating that absolute configuration at this linker position does not significantly influence biological activity (Figure S6). The opposite configuration at the stereocenter on OTX015 was not explored, as prior studies have established its stereochemistry to be critical for BRD4 binding [49]. Next, simple esterification of this hydroxyl group using an azido‐TEG‐COOH linker yielded prodrug ARV825H‐N_3_ for comparison to the esters described above.

**FIGURE 4:**
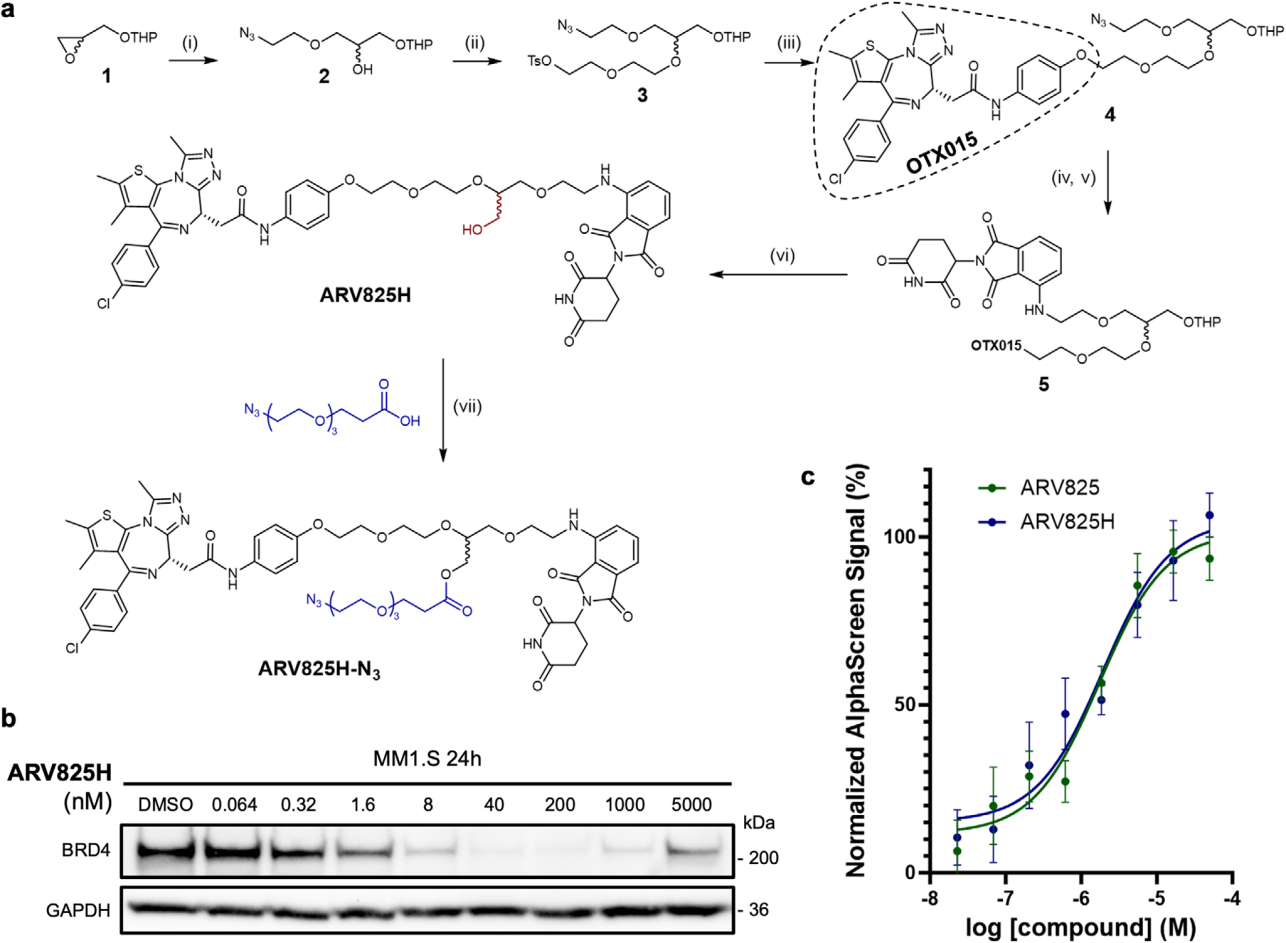
(a) Synthesis of ARV825H‐N_3_. (i) 2‐azidoethanol, NaH, DMF, 0°C; (ii) tos‐PEG2‐Tos, NaH, DMF, 0°C; (iii) OTX015, K_2_CO_3_, MeCN, 80°C; (iv) PPh_3_, MeCN, then H_2_O; (v) 4‐fluorothalidomide, DIPEA, MeCN, 80°C; (vi) TFA/MeOH/H_2_O (3:6:1); (vii) HATU, DIPEA, DCM; (b) Western blot analysis of BRD4 degradation following treatment of KMS12BM cells with ARV825H under various concentrations after 24 h of incubation; (c) AlphaScreen assay to assess ternary complex formation between BRD4, Cereblon, and PROTAC.

### 2.4 Synthesis and Characterization of Branched PROTAC‐Macromonomers (PROTAC‐MMs) and PROTAC‐Bottlebrush Prodrugs (PROTAC‐BPDs)

Having established three strategies for the synthesis of CRBN‐based PROTAC prodrugs, we sought to illustrate the broader versatility of these constructs for conjugation reactions to macromolecular scaffolds. Specifically, we selected BPDs as a representative nanoparticle delivery system to assess the applicability and performance of the prodrugs above under biologically relevant conditions. BPDs are synthesized by ring‐opening metathesis polymerization (ROMP) of norbornene‐based branched macromonomers (MMs) that contain an alkyne group for drug conjugation and a PEG chain to ensure consistent solubility independent of drug identity [50]. Following this general approach, BPDs carrying a wide variety of drugs, drug combinations, and targeting agents can be readily prepared [43, 51, 52, 53, 54]. BPDs display good safety, in vivo efficacy, and favorable pharmacokinetics across multiple payload classes, including E3‐ligase‐binding PROTACs, making them suitable for evaluating the performance of our CRBN‐based PROTAC prodrugs.

Branched MMs of each of the PROTAC prodrugs described above were prepared through copper‐catalyzed azide‐alkyne cycloaddition (CuAAC) “click” reactions between the azide‐containing prodrugs and alkyne‐based 3 kDa PEG‐MM [55]. PROTAC‐BPDs were then prepared via ROMP of each PROTAC‐MM using a target number‐average degree of polymerization of 10 (Figure 5a). PROTAC‐MMs and PROTAC‐BPDs featuring different payloads and linker compositions (Figure 5b) were soluble in water as well as various aqueous buffers. Size‐exclusion chromatography demonstrates efficient conversion of MMs to BPDs in all cases (Figures 5c and S7), while dynamic light scattering revealed similar, narrowly distributed hydrodynamic diameters (*D_h_*) of 10–12 nm (Figure 5d), comparable to typical sizes of BPDs and, for reference, therapeutic antibodies.

**FIGURE 5:**
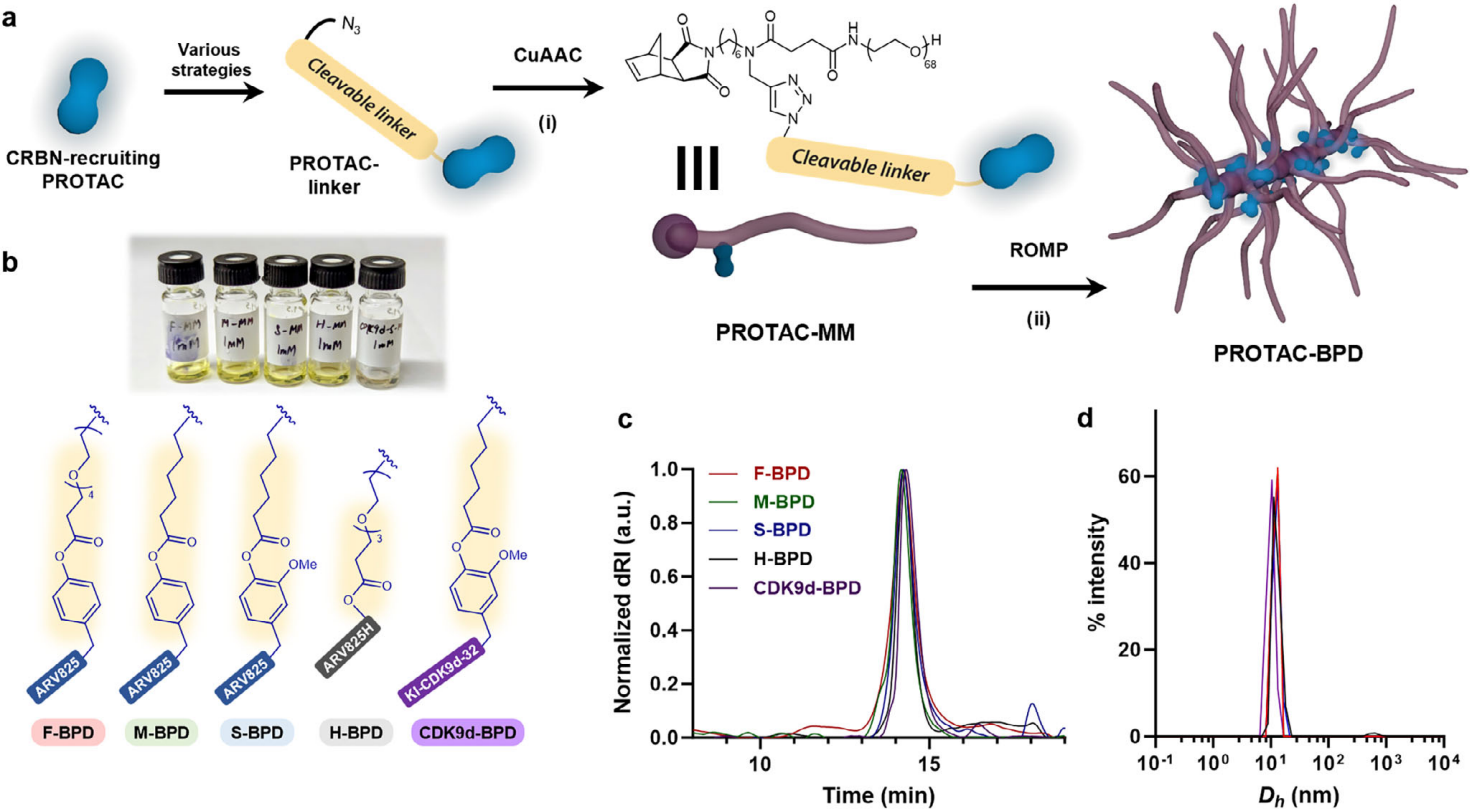
(a) Design and synthesis of PROTAC‐BPDs. (i) PEG‐MM, CuOAc (pinch), DCM/DMF; (ii) Grubbs III generation catalyst (0.1 equiv), THF; then ethyl vinyl ether. (b) Cleavable‐linker structures of various PROTAC‐BPDs synthesized in this study. Macromonomer precursors of the respective BPDs were evaluated for payload release in pH 7.4 PBS at 37 °C, and their release half‐lives quantified. The inset shows fully soluble PROTAC‐MMs in pH 7.4 PBS at 1 mM PROTAC equivalence. (c) Size‐exclusion chromatography traces of PROTAC‐BPDs. (d) *D_h_* of PROTAC‐BPDs determined by dynamic light scattering.

### 2.5 Investigation of PROTAC Release via Ester Linker Hydrolysis

To validate that the ester‐based linkers described above can release unmodified PROTACs via hydrolysis, each of the PROTAC‐MMs was incubated in pH 7.4 PBS at 37 °C; aliquots were collected at defined intervals and the release profile was assessed via liquid chromatography–mass spectrometry (LC‐MS). Under these conditions, ARV825‐MMs exhibited release half‐lives ranging from 9.8 days (F‐MM) to 56 days (S‐MM), while ARV825H‐MM (H‐MM) and CDK9d‐MM exhibited half‐lives of 26.0 and 18.9 days, respectively (Figures 6a and S8). The effect of pH was evaluated at pH 6.0 and 5.5, revealing distinct sensitivities between the para‐benzyl ester and alkyl ester linkers. Addition of esterase further reduced the release half‐lives of F‐MM to ∼5 days and H‐MM to ∼16.6 h (Figures 6b, S9, and S10). The enhanced susceptibility of H‐MM to enzymatic cleavage is consistent with prior studies that observe preferential hydrolysis of less sterically hindered alkyl esters over phenolic esters in the presence of esterases [56, 57]. Slow hydrolysis of the phthalimide functional group of ARV825 was also observed over the course of these studies, which is consistent with prior reports describing the intrinsic lability of this site [58, 59].

**FIGURE 6:**
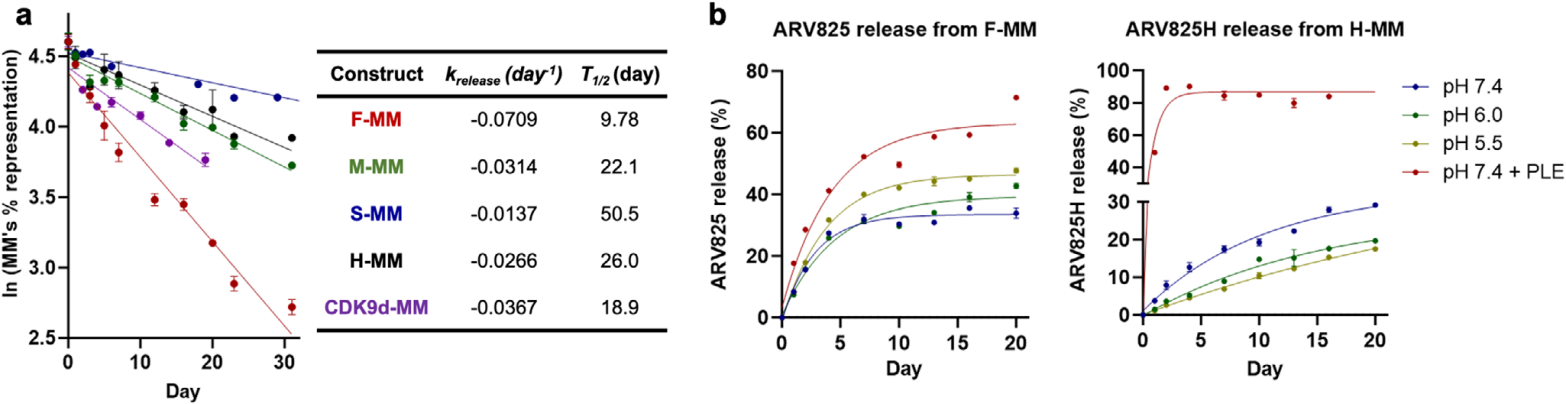
(a) Release kinetics of ARV825 and KI‐CDK9d‐32 from macromonomers with different linker compositions, showing first‐order cleavage behavior. Rate constants (*k*) and corresponding half‐lives are summarized in the table; (b) pH‐ and enzyme‐dependent release profiles of ARV825 from phenyl ester (ARV825‐MM) and alkyl ester (ARV825H‐MM) linkers. The phenyl ester exhibits faster cleavage under acidic conditions but limited response to pig liver esterase (PLE), while the alkyl ester releases more slowly in acid yet undergoes pronounced PLE‐catalyzed cleavage.

### 2.6 Evaluation of PROTAC‐Prodrug Constructs in Multiple Myeloma Cell Models

We next sought to evaluate these PROTAC‐prodrug constructs as degraders of BRD4 and CDK9. Multiple myeloma was chosen as a disease model due to its established dependence on BRD4 and CDK9 signaling for proliferation and survival [60]. We first assessed whether the linker hydrolysis trends observed above translate to corresponding differences in cellular potency in vitro. The F‐, M‐, and S‐MMs and BPDs were evaluated in three multiple myeloma cell lines: MM1.S, EJM, AMO1 (Figures 7a and S11). All constructs produced dose‐dependent decreases in viability after 72 h, with their relative potency following the general trend from the kinetic studies (F‐ > M‐ > S‐), which is consistent with faster‐releasing linkers yielding greater cytotoxicity. The ARV825H‐ and CDK9d‐prodrugs also displayed potent growth inhibition in MM1.S cells, featuring sub‐nanomolar IC_50_ values. Across all constructs, the measured IC_50_ values of BPDs were ∼2–6 fold higher than their MM counterparts, aligning with previous reports that steric shielding in bottlebrush polymers can modulate release kinetics by limiting access to the cleavable linker [61, 62]; however, a deviation from the trend was observed for F‐BPD, which showed slightly enhanced potency compared with F‐MM in all 3 cell lines.

**FIGURE 7:**
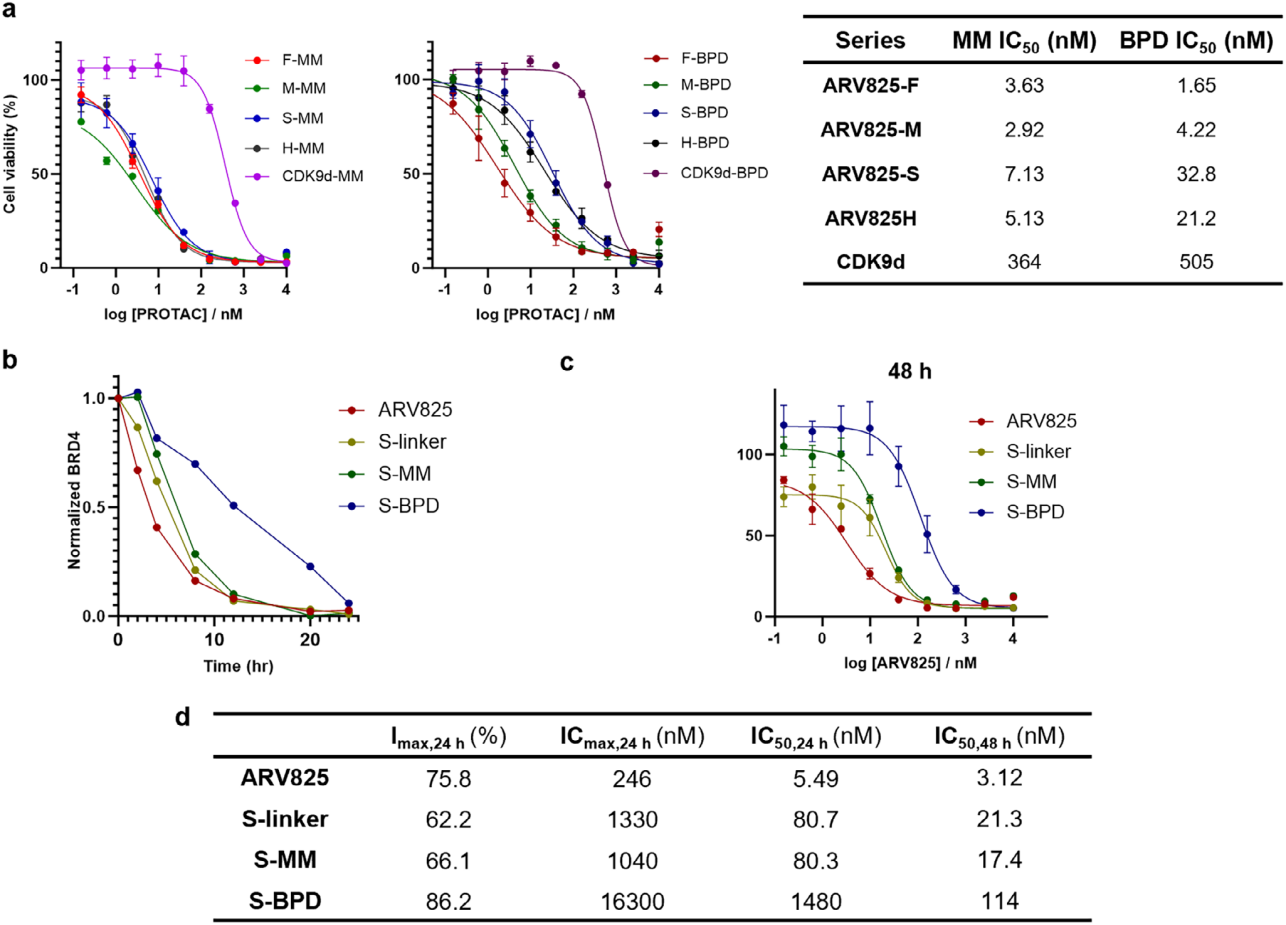
Evaluation of PROTAC–bottlebrush prodrug activity in MM1.S cells. (a) 72 h dose‐response curves and IC_50_ values for MM and BPD constructs synthesized in this study. (b) Time‐dependent degradation of BRD4 following treatment with ARV825 and prodrug constructs S‐linker, S‐MM and S‐BPD. (c) Viability of constructs at 24 and 48 h timepoints. V‐shaped responses at 24 h were fitted with the hook model (Note S2), while 48 h data follow a classical sigmoidal dose‐response curve. (d) Extracted kinetic parameters (*I_max_, IC_max_, IC_50_*) from the fitted 24 and 48 h datasets, showing time‐dependent enhancement in potency and maximal response. Data are shown as mean ± SD from *n* = 3 biologically independent wells.

To characterize the influence of each of the stepwise modifications made to the PROTACs above, that is, PROTAC→ PROTAC azide prodrug → PROTAC‐MM → PROTAC‐BPDs, we compared the BRD4 expression and cytotoxicities of ARV825, ARV825‐S‐N_3_, S‐MM, and S‐BPD. Time‐course studies revealed a progressively delayed onset of BRD4 degradation with each conjugation (Figure 7b). Within 2 h, ARV‐825 and ARV825‐S‐N_3_ achieved degradation of ∼33% and ∼14% of total BRD4, respectively, while BRD4 levels remained unchanged for S‐MM and S‐BPD. At 12 h, this value grew to 90% for all constructs except S‐BPD where 50% of total BRD4 remained. Complete degradation was observed for all constructs at 24 h.

Cell viability assays using the above constructs at 12, 24, and 48 h displayed the same ordered progression. While minimal cytotoxicity was observed at 12 h, V‐shaped dose–response curves were observed at 24 h (Figure 7c). Although the hook model is more commonly described for quantifying protein degradation, we can consider viability as a downstream pharmacodynamic response to define, for this study, values of *I_max_* (maximal inhibition observed), *IC_max_* (concentration at *V_min_*), and *IC_50_* (concentration at half *I_max_* inhibition). By adapting the hook model described by Haid and Reichel [63] (Note S2), the 24 h data were be fitted to obtain values of *IC_50_* and *IC_max_*, showing clear differences in potency following trends of ARV825 > S‐linker ≈ S‐MM > S‐BPD. By 48 h, viability profiles within the assayed range of concentrations can be fully fitted to sigmoidal dose‐response curves, with trends mirroring those observed at 24 h. These results collectively show that as ARV825 is converted to increasingly larger constructs, the onset of BRD4 degradation and consequently cell growth inhibition is delayed, yet full activity is achieved following PROTAC release and incubation for sufficient time.

Having established the kinetic and potency relationships among the tiered constructs of ARV825, we next examined dose‐dependent target degradation using BRD4‐degrading (S‐BPD, H‐BPD) and CDK9‐degrading (CDK9d‐BPD) constructs. In each case, the prodrugs exhibited clear, concentration‐dependent depletion of their respective proteins after 24 h in MM1.S cells (Figure 8a). The extent of degradation increased proportionally with dose, reaching near‐complete loss at 300 nM for S‐BPD and H‐BPD, and at 100 nM for CDK9d‐BPD.

**FIGURE 8:**
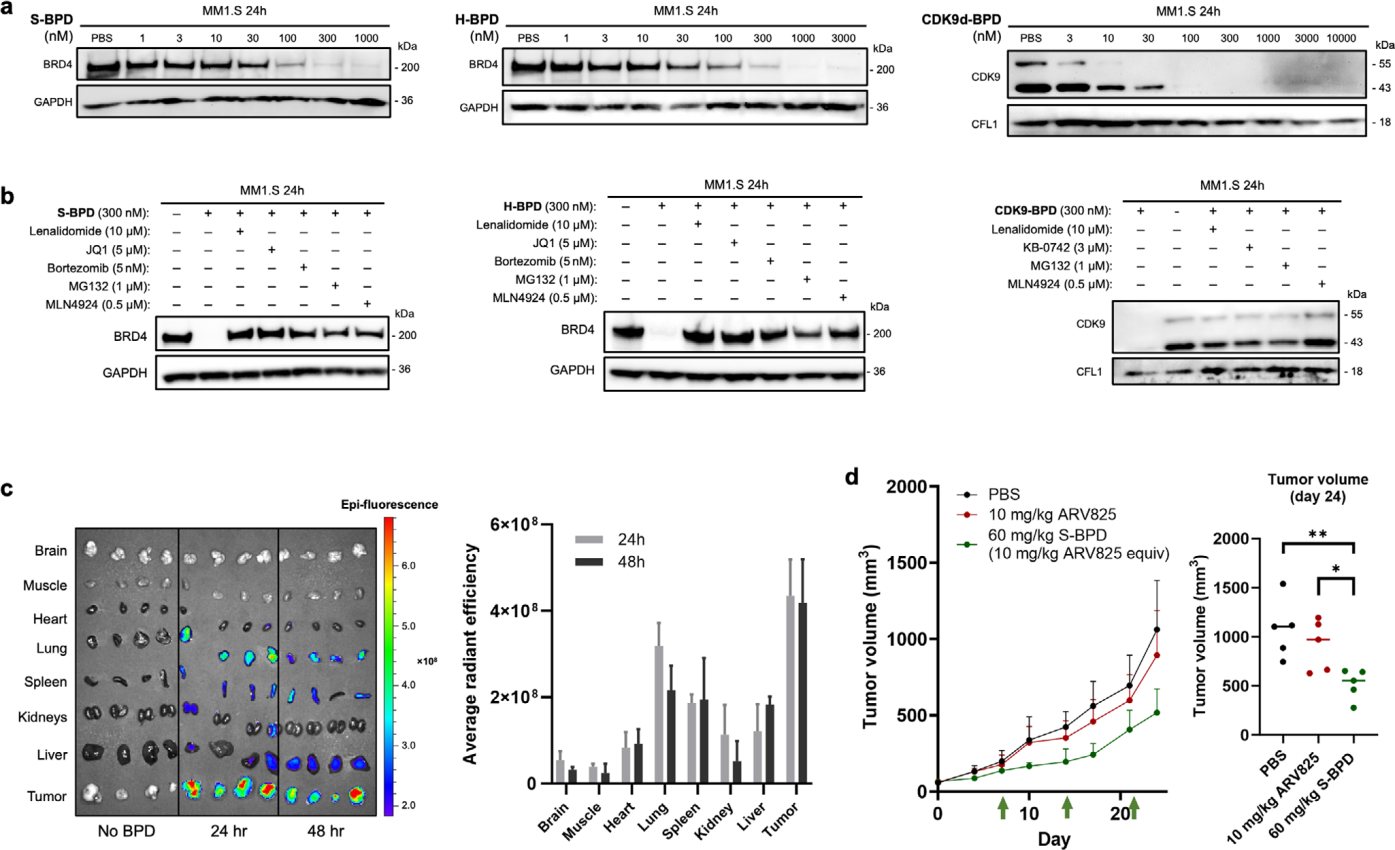
(a) Dose‐dependent degradation of BRD4 by S‐BPD/H‐BPD, and degradation of CDK9 by CDK9d‐BPD. (b) Inhibitor‐rescue assays confirm that protein degradation induced by PROTAC‐BPDs proceeds through ternary complex formation followed by the canonical ubiquitin‐proteasome system. (c) Representative fluorescence images and quantitative biodistribution analysis show preferential accumulation of the Cy7‐S‐BPD in tumor tissue. (d) Tumor growth curves following i.p. administration of S‐BPD or free ARV825, showing delayed tumor progression in the BPD‐treated group. Arrows indicate dosing days. Tumor growth data are shown as mean ± SD (*n* = 5 mice per group). Statistical significance was determined by ordinary one‐way ANOVA with Šídak's multiple‐comparisons test; *p < 0.05, **p < 0.01.

To validate the mechanism of the observed degradation, inhibitor‐rescue experiments were conducted on S‐BPD, H‐BPD and CDK9d‐BPD (Figures 8b and S12). BRD4 degradation was fully suppressed by proteasome inhibitors (Bortezomib, MG132) and by NEDD8‐activating enzyme inhibitor (MLN4924), confirming dependence on the ubiquitin–proteasome system. Competitive inhibition with the CRBN ligand (lenalidomide) and BRD4 inhibitor (JQ1) similarly restored BRD4 levels, supporting the relevance of ternary complex formation in its mechanism of action. Likewise, CDK9 degradation with CDK9d‐BPD was rescued with a competitive inhibitor of CDK9, KB0742, alongside the other aforementioned inhibitors. Together, these results support a mechanism in which linker cleavage regenerates the parent PROTAC, enabling recruitment of a ternary complex with CRBN, and eventual downstream proteasomal degradation that underlies the observed cytotoxic response.

### 2.7 Preliminary In Vivo Evaluation

To explore the translational potential of these PROTAC prodrugs, we performed a preliminary assessment of tumor accumulation and antitumor activity using the S‐BPD construct in a subcutaneous KMS‐12‐BM multiple myeloma model. To facilitate fluorescence imaging, we synthesized a Cy7‐S‐BPD (Figure S13) that preserves the physicochemical and release characteristics of S‐BPD. In vivo imaging revealed preferential accumulation of the BPD within tumor tissue (Figure 8c), consistent with the enhanced tumor retention phenomenon observed for BPDs carrying different drugs [43, 54]. Finally, S‐BPD treatment led to a statistically significant delay in tumor growth relative to the parent PROTAC in a subcutaneous KMS‐12‐BM tumor model (Figure 8d), suggesting that controlled release and BPD‐mediated delivery may contribute to sustained activity in vivo (Figure S14). These results suggest that the PROTAC prodrug linker syntheses introduced here are promising for future translational development.

## 3 Conclusions

Herein we establish three complementary strategies that address synthetic barriers to covalent conjugation of traceless cleavable linkers to CRBN‐recruiting PROTACs. By leveraging a triazole quaternization approach, an amine quaternization approach, or the installation of a minimal hydroxyl handle along the linker, we expand the chemical space of accessible PROTAC prodrugs within these scaffolds without compromising their CRBN‐binding motifs. Furthermore, these conjugation reactions feature simple procedures and yields exceeding other reported strategies, providing an orthogonal toolkit for functionalizing CRBN‐recruiting PROTACs.

Applying these reactions, we synthesized a family of ARV825‐ and KI‐CDK9d‐32‐derived PROTAC‐prodrugs encompassing three levels of conjugation: PROTAC‐azides, PROTAC‐MMs, and PROTAC‐BPDs. Using these constructs, we examined how increasing conjugation affects downstream pharmacologic behavior for representative constructs, including the timing and extent of protein degradation and corresponding cytotoxicity. Across all constructs, hydrolysis rate correlated with cellular potency, demonstrating that chemical control of linker cleavage translates into predictable biological outcomes. Mechanistic studies using representative examples of each linker type confirmed that degradation proceeds through CRBN engagement and the ubiquitin–proteasome system, and that the degradation profiles of the BPDs closely mirror those of the parent PROTACs. Collectively, these results support a unified chemical framework for designing PROTAC prodrugs with programmable activation and pharmacological timing. A preliminary in vivo study using one PROTAC‐BPD also demonstrated favorable tumor accumulation and antitumor activity, motivating future studies of a broader range of these molecules in the future.

Looking forward, the PROTAC‐prodrugs developed here feature an azide handle for conjugation to a wide range of delivery scaffolds, including polymeric, lipid, and biomolecular carriers, offering opportunities to couple controlled PROTAC release with established targeted delivery technologies, potentially addressing current limitations in pharmacokinetics and tissue selectivity. Continued development along these lines could extend chemically encoded control of degradation beyond the BPDs demonstrated here, paving the way for new strategies in the rational design of CRBN‐PROTAC prodrugs for clinical translation.

## Author Contributions

**Aiden X. Wang**: conceptualization, investigation, writing – original draft, methodology, validation, visualization, writing – review and editing, formal analysis, data curation. **Bin Liu**: investigation, methodology, writing – review and editing, validation. **Elise M. Ackerman**: writing – review and editing, methodology, investigation, validation. **Yichen Xiang**: investigation, methodology, writing – review and editing, validation. **Hung V.‐T. Nguyen**: investigation, methodology, writing – review and editing, validation. **Yivan Jiang**: investigation, methodology, validation, writing – review and editing. **Angela N. Koehler**: writing – review and editing, funding acquisition, supervision, project administration. **Jeremiah A. Johnson**: conceptualization, funding acquisition, writing – original draft, writing – review and editing, project administration, supervision.

## Funding

This work was supported by the NIH (2R01CA220468‐06A1), the CCSG Award for the Koch Institute (P30‐CA014051), the Yosemite–American Cancer Society Award (YACS‐24‐1343208‐01‐YACS), and the Koch Institute Frontier Research Program through the Kathy and Curt Marble Cancer Research Fund. A. X. W. acknowledges support from the Koch Institute Graduate Fellowship Program.

## Conflicts of Interest

The authors declare the following competing financial interest(s): A.X.W., H.V.T.N., Y.J., and J.A.J. are named inventors on patents filed on this work.

## Supporting information



The authors have cited additional references within the Supporting Information [43, 44, 55, 59, 63, 64, 65, 66, 67, 68] **Supporting File**: anie73907‐sup‐0001‐SuppMat.docx.

## Data Availability

The data that support the findings of this study are available from the corresponding author upon reasonable request.
