# Supplementary material for: Generalizable Strategies for the Synthesis of Cereblon‐Recruiting PROTAC Prodrugs
Source: Angew Chem Int Ed Engl. 2026 Aug 5;65(40):e3238473. doi: 10.1002/anie.3238473 (PMC13618278; doi:10.1002/anie.3238473)
Supplement: Supplementary file 1 [file ANIE-65-e3238473-s001.docx]

*Supporting information for:*

**Generalizable Strategies for the Synthesis of Cereblon-Recruiting PROTAC Prodrugs**

Aiden X. Wang,^1^ Bin Liu,^1^ Elise M. Ackerman,^1^ Yichen Xiang,^2^ Hung, V.-T. Nguyen,^1^ Yivan Jiang,^1^ Angela N. Koehler,^2,3,4,5^ Jeremiah A. Johnson^1,2^*

**Affiliations**

^1^Department of Chemistry, Massachusetts Institute of Technology, 77 Massachusetts Avenue, Cambridge, MA 02139, U.S.A.

^2^Koch Institute for Integrative Cancer Research, Massachusetts Institute of Technology, Cambridge, MA 02139

^3^Broad Institute of MIT and Harvard, Cambridge, MA, 02142, USA

^4^Department of Biological Engineering, Massachusetts Institute of Technology, Cambridge, MA, 02139, USA

^5^Center for Precision Cancer Medicine, Massachusetts Institute of Technology, Cambridge, MA, 02139, USA

*Correspondence to: [jaj2109@mit.edu](mailto:jaj2109@mit.edu)

Table of Contents

[Section A: Materials, Methods and Instrumentation	3](#_Toc219394115)

[Section B: Supplementary text and figures	5](#_Toc219394116)

[**Figure S1**: Conjugation of ARV825 through triazole	5](#_Toc219394117)

[**Figure S2:** ^1^H-^13^C HMBC analysis of ARV825-S-linker	6](#_Toc219394118)

[**Figure S3:** 2D NMR analyses of CDK9d-linker	7](#_Toc219394119)

[**Figure S4:** Non-binding role of linker in analogous PROTACs	8](#_Toc219394120)

[**Figure S5:** Degradation of BRD4 in multiple myeloma cells by ARV825H	8](#_Toc219394121)

[**Figure S6:** Characterization of BPDs	9](#_Toc219394122)

[**Figure S7:** Traces of in vitro PROTAC release from MMs (1 mg/mL initial MM)	10](#_Toc219394123)

[**Figure S8:** Traces of in vitro ARV825 release from F-MM (5 mg/mL initial MM)	11](#_Toc219394124)

[**Figure S9:** Traces of in-vitro ARV825H release from H-MM (5 mg/mL initial MM)	12](#_Toc219394125)

[**Figure S10:** 72 h Viability of MM and BPD constructs in multiple myeloma cell lines	13](#_Toc219394126)

[**Figure S11:** Protein abundance analysis of inhibitor rescue of protein degradation by BPDs.	14](#_Toc219394127)

[**Figure S12:** Synthesis of Cy7-S-BPD	15](#_Toc219394128)

[**Figure S13:** Acute toxicity assessment of ARV825-S-BPD in BALB/c mice*.*	15](#_Toc219394129)

[**Supplementary note 1:** Identification of distinct species in PROTAC release studies	16](#_Toc219394130)

[**Supplementary note 2:** Hook curve parameters used to fit Fig 7c.	19](#_Toc219394131)

[Section C: Synthetic Procedures	20](#_Toc219394132)

[1. Synthesis of PROTAC-linkers, PROTAC-MMs, and PROTAC-BPDs	20](#_Toc219394133)

[1.1. Synthesis of ARV825-S-BPD	20](#_Toc219394134)

[1.2. Synthesis of ARV825-M-BPD	25](#_Toc219394135)

[1.3. Synthesis of ARV825-F-BPD	30](#_Toc219394136)

[1.4. Synthesis of CDK9d-BPD	32](#_Toc219394137)

[1.5. Synthesis of ARV825H-BPD	35](#_Toc219394138)

[1.6. Synthesis of Cy7-ARV825-S-BPD	37](#_Toc219394139)

[2. Synthesis of ARV825H	38](#_Toc219394140)

[NMR Spectra	44](#_Toc219394141)

[MALDI-MS Spectra	61](#_Toc219394142)

[**References**	71](#_Toc219394143)

# Section A: Materials, Methods and Instrumentation

All reagents were purchased from commercial suppliers and were used without further purification unless otherwise stated. ARV825 was purchased from MedChemExpress or Adooq Bioscience, OTX-015 was purchased from Amadis Chemical, NH_2_-PEG3K-OH was purchased from Xiamen SINOPEG Biotech Co., Ltd; Grubbs 3^rd^ generation bis-pyridyl catalyst G3 ^[64]^, linker **1** ^[43]^**,** and alkyne macromonomer ^[55]^ were prepared as previously reported. KI-CDK9d-32 was synthesized according to procedures previously reported ^[65]^, starting from KI-ARV03 ^[66]^. Column chromatography was carried out on silica gel 60F (EMD Millipore, 0.040–0.063 mm) or on aluminum oxide (Sigma-Aldrich, activated, neutral, Brockmann Activity I).

**Chromatography.** LC-MS Measurements were performed on an Agilent 1260 LC system equipped with a Zorbax SB-C18 rapid resolution HT column, using a binary solvent system (MeCN and H2O with 0.1% CH3COOH). Recycling preparative HPLC was performed on a LaboACE system (Japan Analytical Industry), using a JAIGEL-2 HR JAIGEL-2.5HR column in series. Size Exclusion Chromatography (SEC) analyses were performed on an Agilent 1260 Infinity setup with two Agilent PL1110-6500 columns in tandem and a 0.025 M LiBr DMF mobile phase run at 60 °C. The differential refractive index (dRI) of each sample was monitored by using a Wyatt Optilab T-rEX detector; the static light scattering (LS) signal was acquired with a Wyatt Dawn Heleos-II detector. SEC traces are normalized to the highest peak value for each sample.

**Nuclear magnetic resonance (NMR) spectroscopy.** Spectra were recorded on a Bruker AVANCE III-400 spectrometer with working frequencies of 400 (^1^H) and 100 (^13^C) MHz or on an AVANCE-600 spectrometer with working frequencies of 600 (^1^H) and 151 (^13^C) MHz. Chemical shifts are reported in ppm relative to the signals corresponding to the residual non-deuterated solvents: CDCl_3_: δ_H_ = 7.26 ppm and δ_C_ = 77.16 ppm; MeCN: δ_H_ = 1.94 ppm and δ_C_ = 1.32, 118.26 ppm.

**Mass spectrometry.** High-resolution mass spectra were measured on a JEOL AccuTOF LC-Plus 4G with an IonSense DART or an Agilent 6546 Q-Tof mass spectrometer. MS/MS analysis was performed using an Agilent 6546 Q-Tof mass spectrometer. Matrix-assisted laser desorption/ionization time-of-flight (MALDI-TOF) analyses were collected on a Bruker OmniFlex instrument, using either α-Cyano-4-hydroxycinnamic acid (CHCA) or trans-2-[3-(4-tert-Butylphenyl)-2-methyl-2-propenylidene] (DCTB) as the matrix.

**Dynamic light scattering (DLS).** Measurements were performed on a Wyatt Technology Mobius DLS instrument. Nanoparticle suspensions were prepared in a solution of nanopure water (MilliQ) at a concentration of ~1 mg/mL. The resulting suspensions were passed through a 0.45 μm Nalgene filter (PES membrane) into quartz cuvettes, which were pre-cleaned with compressed air. Measurements were made in sets of 10 acquisitions; the average hydrodynamic diameters were calculated by using the DLS correlation function via a regularization fitting method (Dynamics 7.4.0.72 software package from Wyatt Technology).

**Alphascreen assay.** GST-tagged BRD4 [BD1+BD2] (60 nM, BPS Bioscience, #31044) and His-tagged Cereblon/DDB1/CULA4/RBX1 (30 nM, BPS Bioscience, #110329-1) were incubated with serially diluted ARV825 or ARV825H for 30 min in opaque white 384-well plates. The buffer used in the assay comprised 25 mM HEPES (pH 7.4), 100 mM NaCl, 0.1% BSA, and 0.05% Tween 20. Nickel chelate Alpha donor beads (25 µM final concentration, BPS Bioscience, #6760619C) and GST acceptor beads (25 µM final concentration, BPS Bioscience, #6760603C) were then added to each well, mixed, and incubated for 1hr in the dark at r.t. Luminescence detection was performed on a Tecan Spark plate reader (ex680/em570).

**In vitro drug release studies.** In a 2 mL vial, PROTAC-conjugated macromonomers were dissolved at a concentration of 1 or 5 mg/mL (~19% PROTAC by mass) in respective buffer solutions. The vials were placed in a temperature-controlled incubator set at 37 °C. At various times, 20 μL aliquots were withdrawn and thoroughly mixed with a 0.05 mg/mL solution of salicylic acid in DMSO as the internal standard. Quantification was performed using UV absorbance at 254 nm. Analytical LC-MS conditions: 5%–30% MeCN/H_2_O [0–2min], 30%–70% MeCN/H_2_O [2–15 min], 70%–100% MeCN/H_2_O [15–17 min], ZORBAX C18 column. Data are presented as mean ± standard deviation (SD) for n = 3 unless otherwise stated. Drug release half-lives were determined by non-linear regression by fitting a one-phase association or dissociation curve.

**Cell culture.** All cell lines were confirmed to be mycoplasma free by the MycoAlert® Mycoplasma Detection Kit (Lonza) and authenticated using ATCC’s STR authentication service. KMS12BM, KMS11, MM.1S, EJM and AMO1 cells were grown in RPMI 1640 Medium supplemented with 10% FBS and 1X Ab-Am. All cells were maintained at 37 °C in 5% CO2. Cells were passaged when approximately 80% confluent at 1:3 split ratio after detachment. KMS11 and MM.1S cells contained a mix of adherent and suspension cells; their adherent cells were detached by incubating in TrypLE Express (Thermo Fisher Scientific, 12604039) for 5 minutes or by scraping. All centrifugation steps were at 300 x g for 5 min.

| **Cell line** | **RRID** | **Source** |
| --- | --- | --- |
| KMS12BM | CVCL_1334 | Cytion |
| KMS11 | CVCL_2989 | Gift from Ghobrial Lab (DFCI) |
| MM1.S | CVCL_8792 | ATCC |
| EJM | CVCL_2030 | Gift from Ghobrial Lab (DFCI) |
| AMO1 | CVCL_1806 | Gift from Ghobrial Lab (DFCI) |

**Western blotting.** KMS11, KMS12BM, or MM1.S cells were plated on 24-well plates at a density of 8 x 10^5^ cells/mL. Compounds at different concentrations or DMSO/PBS were added to the plates and left to incubate for either 24 h or otherwise stated. Cells were then scraped and washed twice with ice-cold PBS with centrifugation at 2500 x g for 5 min. After discarding the supernatant, RIPA buffer (Thermo Scientific™, Cat#89900) was added to lyse the cells. The cell lysis solution was then denatured at 95 °C and separated with 3-8% Tris-Acetate SDSPAGE (Thermo Scientific™, Cat#TA03810BOX). Proteins were transferred to a PVDF membrane (Invitrogen™, CAT#LC2002). Primary antibodies for BRD4 (CST, #E2A7X, Rabbit), CDK9 (CST, #C12F7, Rabbit), GAPDH (CST, #14C10, Rabbit), CFL1 (CST, #D3F9, Rabbit) and anti-Rabbit secondary antibody (CST, #7074P2, HRP-linked) were used to bind proteins. HRP substrate (SignalFire ECL, CST, #6883) was used for chemiluminescent detection, photographed using BioRad ChemiDoc imager, and processed with ImageJ.

**Viability studies.** Exponentially growing cells were plated at 25000 cells per well in 96 well plates in 90 μL medium. MM and BPD samples were prepared in PBS and filtered through a 0.22 μm PES filter to remove any trace free PROTAC, while free PROTAC and PROTAC-linkers were prepared in DMSO. 10 μL of serially diluted compounds were then added to each well (final DMSO concentration never exceeding 0.1%). After incubation for 72 h (unless stated otherwise) at 37 °C, cells were assayed using the CCK8 assay from MedChemExpress: add 10 μL CCK8 reagent, shake gently and incubate at 37 °C for 3 h. The plate was then read at 450 nm using a SpectraMax M3 plate reader.

For analysis of relative viability, to correct plate-to-plate variability, we set each raw result relative to the maximum readout within each plate (maximum-corrected values). For each treatment-dose condition, the normalized value was calculated by dividing the maximum-corrected value by the mean of the maximum-corrected responses from media-only wells on the same plate. Data are presented as mean ± standard deviation (SD) for n = 3 unless otherwise stated. Dose-response curves and half-maximal inhibitory concentration (IC_50_) values were generated by nonlinear regression in GraphPad Prism by fitting a 4-parameter logistic curve. For viability assays that exhibited V-shaped dose-response behavior, data were fitted using the adapted hook-effect model in Supplementary Note 2.

**Animal studies.** Animal usage: All experiments involving animals were reviewed and approved by the MIT Committee for Animal Care (CAC). In vivo tumor imaging of the subcutaneous model (KMS12BM; SCID-beige (female), Charles River) was performed at the Imaging Facility Center of the David H. Koch Institute for Integrative Cancer Research at MIT. Animals received an alfalfa-free diet (TestDiet) at least 2 weeks before the start of the studies to minimize auto-fluorescence. No estrogen was used for these experiments. BALB/c mice (female, 4–6 weeks old, Taconic; n = 5 mice/group) were used for toxicity studies.

**In vivo efficacy studies.** KMS12-BM cells were cultured following the protocol described above to a final confluency of 80%. Cells were harvested and then mixed with Geltrex (ThermoFisher, #A1413201) and sterile pH 7.4 PBS buffer (1 : 1), and injected subcutaneously (8.0 × 10^6^ cells, 0.1 mL) into the right flank of SCID-beige mice. After 14 days, tumor sizes (volume = π/6(L×W×H)) were measured with digital calipers and mice were randomized into treatment groups with average tumor size of 60 mm^3^. Tumor-bearing mice were then given intraperitoneal injections of 0.1 mL of samples at varying concentrations every 7 days. Mouse weights and tumor volume were monitored and recorded twice a week for 4 weeks until appropriate end-points for euthanasia were reached. Tumor growth data are shown as mean ± SD (n = 5 mice per group). Statistical significance was determined by ordinary one-way ANOVA with Šídak’s multiple-comparisons test in GraphPad Prism.

# Section B: Supplementary text and figures

**Figure S1**: Conjugation of ARV825 through triazole

Above: Conjugation reaction performed under initial conditions of 60 °C. NMR time course over 70 h tracks the shifts in the benzyl peak (4.58 ppm 🡪 5.48ppm) and 3x methyl peaks (2.60, 2.39, 1.65 ppm 🡪 2.96, 2.48, 1.70 ppm). Below: LCMS analysis of purified ARV825-S-N_3_

## **Figure S2:** ^1^H-^13^C HMBC analysis of ARV825-S-linker

Carbons **1** and **2** were labelled on the HMBC spectra based on their expected resonances with protons **2** and **3**/**4** respectively. Presence of **C1** – **H1** resonance and absence of **C2** – **H1** resonance suggests that triazole conjugation occurs on the less hindered nitrogen

## **Figure S3:** 2D NMR analyses of CDK9d-linker

^1^H NOESY ascertains the proximity of protons **3** and **4**, and piperazine protons **1** and **2**. ^1^H–^13^C HMBC identifies cross resonances for carbons **2** and **3** with the other carbon’s attached proton (**C2** – **H4**, **C3** – **H3**).

## **Figure S4:** Non-binding role of linker in analogous PROTACs

Binding poses of PROTACs dBET6 (left), dBET23 (middle) and CFT1297 (right) in crystallized ternary complexes with BRD4 and Cereblon suggests a non-binding role of the linker region (blue). Empty space available along the linker suggests it is the optimal region to accommodate a small conjugation handle. Images were rendered using Pymol 3.1 with structures 6BOY (dBET6), 6BN7 (dBET23) and BRQ9 (CFT1297) obtained from the Protein Data Bank.

|  | ***D_max_*** *(%)* | ***DC_max_*** *(nM)* | ***DC_50_*** *(nM)* |
| --- | --- | --- | --- |
| **KMS11** | 76.7 | 314 | 23.6 |
| **KMS12BM** | 100 | 44.8 | 3.76 |
| **MM1.S** | 97.9 | 126.3 | 1.25 |

## **Figure S5:** Degradation of BRD4 in multiple myeloma cells by ARV825H

Western blotting of cell lysates of KMS11 / MM1.S cells after 24 h incubation with ARV825H. Complete degradation of BRD4 can be observed in MM1.S cells at 40 nM. Degradation efficacy of BRD4 of ARV825H closely resembles that of ARV825 reported in literature ^[67]^.

## **Figure S6:** *In-vitro* characterization of a stereochemically pure variant of ARV825H

A stereochemically pure variant of ARV825H was synthesized from (+)-glycidol using procedures for ARV825. Cytotoxicity was evaluated in KMS11 cells, revealing comparable IC_50_ values for the stereochemically pure and racemic variants.

*Đ* = 1.07

*Đ* = 1.16

*Đ* = 1.14

*Đ* = 1.14

*Đ* = 1.03

## **Figure S7:** Characterization of BPDs

Size-exclusion chromatograms for PROTAC-BPDs and their MM starting materials. Dispersity (*Đ*) values for respective BPDs are provided on each graph.

| **MM** | ***k*** | ***t*_1/2_ (day)** |
| --- | --- | --- |
| F-MM | -0.0709 | 9.78 |
| M-MM | -0.0314 | 22.1 |
| S-MM | -0.0137 | 50.5 |
| H-MM | -0.0266 | 26.0 |

## **Figure S8:** Traces of in vitro PROTAC release from MMs (1 mg/mL initial MM)

Top: Relative concentration of species present in samples during incubation (detailed structures and characterization in Supplementary note 1). Macromonomer concentrations are normalized to UV signal at *t* = 0, while ARV825 and ARV825_Hyd are normalized to a sample of ARV825 at theoretical max concentration (100% release).

Middle: Species are grouped according to the following and fitted with a one-phase exponential decay/association curve:

- MMs	= [ARV825-MM] + [ARV825-Hyd-MM]
- SMs	= [ARV825] + [ARV825_Hyd]
- ARV825’s	= [ARV825-MM] + [ARV825]
- ARV825_Hyd’s	= [ARV825-Hyd-MM] + [ARV825-Hyd]

Bottom: Combined plots comparing decrease in drug-conjugated MMs (proxy for linker cleavage) and their fitted rate constants. Rate constants follow trends in electron density around phenyl ester functional group reported previously ^[44]^.

| **Conditions** | ***k*** | ***t*_1/2_ (day)** |
| --- | --- | --- |
| pH 7.4 | -0.0711 | 9.75 |
| pH 6.0 | -0.0811 | 8.55 |
| pH 5.5 | -0.106 | 6.55 |
| pH 7.4 + PLE | -0.134 | 5.18 |

## **Figure S9:** Traces of in vitro ARV825 release from F-MM (5 mg/mL initial MM)

ARV825-F-MM were prepared at 5 mg/mL in buffers at pH 7.4 (PBS), pH 6.0 (Citrate), or pH 5.5 (Acetate). Pig liver esterase (PLE) was added to a pH 7.4 sample at a final activity of 1U/mL.

The *para*-benzyl ester linker demonstrates accelerated rate of cleavage from pH 7.4 to pH 5.5, with a nearly 2-fold rate acceleration under PLE conditions.

| **Conditions** | ***k*** | ***t*_1/2_ (day)** |
| --- | --- | --- |
| pH 7.4 | -0.0264 | 26.1 |
| pH 6.0 | -0.0142 | 48.8 |
| pH 5.5 | -0.0138 | 50.2 |
| pH 7.4 + PLE | -0.809 | 0.857 |

## **Figure S10:** Traces of in-vitro ARV825H release from H-MM (5 mg/mL initial MM)

ARV825-H-MM were prepared at 5 mg/mL in buffers at pH 7.4 (PBS), pH 6.0 (Citrate), or pH 5.5 (Acetate). Pig liver esterase (PLE) was added to a pH 7.4 sample at a final activity of 1 U/mL.

The alkyl ester linker demonstrates slight decelerated rate of cleavage from pH 7.4 to pH 5.5, with a ~30-fold rate acceleration under PLE conditions.

## **Figure S11:** 72 h Viability of MM and BPD constructs in multiple myeloma cell lines

Viability studies of all synthesized MM and BPD constructs in multiple myeloma cell lines MM1.S, EJM, and AMO1. Assays were run in 96 well plates and quantified using CCK8 assay.

## **Figure S12:** Protein abundance analysis of inhibitor rescue of protein degradation by BPDs.

Western blot analysis showing degradation (BRD4 by S-BPD/H-BPD, CDK9 by CDK9d-BPD) and rescue assessments in MM1.S after 24h exposure. Concentrations used: All BPDs (300 nM equivalent payload); Lenalidomide (10 µM); JQ1 (5 µM); KB0742 (3 µM); Bortezomib (5 nM); MG132 (1 µM); MLN4924 (0.5 µM).

## **Figure S13:** Synthesis of Cy7-S-BPD

Cy7-S-BPD was synthesized via ROMP of S-MM incorporated with 5% Cy7-MM, achieving statistical copolymerization of the Cy7-MM into the final BPD

## **Figure S14:** Acute toxicity assessment of ARV825-S-BPD in BALB/c mice.

Body weight change was monitored over 7 days following intraperitoneal administration of ARV825-S-BPD at doses of 15, 30, or 60 mg kg⁻¹, or control bottlebrush polymer (BBP, 120 mg kg⁻¹). No significant weight loss or adverse effects were observed across all treatment groups compared with PBS, indicating good short-term tolerability of the ARV825-BPD formulation. Error bars represent mean ± SD (n = 5 per group)

## **Supplementary note 1:** Identification of distinct species in PROTAC release studies

In all PROTAC-MMs, we found the hydrolytic release of each PROTAC from its macromonomer precursor to proceed through a sequence of intermediates (see below).

These species (except ARV825-Hyd^2^ which was only detected after 6 months of incubation) can be clearly identified in the LC-MS traces

ARV825-hyd and ARV825-hyd² can be positively identified using MS and MS/MS analysis, revealing that hydrolysis occurs predominantly at the phthalimide moiety of the pomalidomide substructure, consistent with prior reports describing intrinsic lability at this position ^[59,68]^.

Identifying the site of hydrolysis in ARV825-hyd by MS/MS (below)

Hydrolysis of ARV825 can occur at the phthalimide or glutarimide groups, both generating species of mass 940.30. MS/MS was performed to verify the site of hydrolysis, revealing a fragment of mass ~813.25 only present in ARV825-Hyd and ARV825-Hyd^2^. If 1^st^ hydrolysis occurs on phthalimide (red arrow), the 2^nd^ hydrolysis would be unable to generate the 813.25 mass fragment under MS/MS (absent for ARV825), supporting the hypothesis that hydrolysis occurs predominantly on phthalimide (green arrow).

Similar species are observed from release study of ARV825H-MM and CDK9d-MM

## **Supplementary note 2:** Hook curve parameters used to fit Fig 7c.

Haid and Reichel ^[63]^ proposed the following equation describing the hook model:

$$D\left( C \right)=D_{max}\times\frac{{DC}_{50}^{2}+{DC}_{max}^{2}-2\times{DC}_{50}\times{DC}_{max}}{{DC}_{50}^{2}+{DC}_{max}^{2}-4\times{DC}_{50}\times{DC}_{max}+{DC}_{50}\times C+\frac{{DC}_{50}\times{DC}_{max}^{2}}{C}}$$

Where, DC_max_ = PROTAC concentration at maximum degradation efficacy; DC_50_ = PROTAC concentration (< DC_max_) where half-maximum degradation efficacy is achieved; D_max_ = maximum degradation of target protein.

The inverse curve can be obtained by subtracting the entire above expression to another fitted constant Top, which indicates the max value of analyzed protein. The equation entered into GraphPad Prism is then:

Y =	Top - D_max_ * (DC_50_^2 + DC_max_^2 - 2*DC_50_*DC_max_) / (DC_50_^2 + DC_max_^2 - 4*DC_50_*DC_max_ + DC_50_*X + (DC_50_*DC_max_^2)/X)

With initial values Top = 100, DC_max_ = concentration at dip, D_max_ = Protein level at dip, DC_max_ < DC_50_

Through fitting, we obtain:

- IC_50_ = DC_50_
- IC_max_ = DC_max_
- I_max_ = D_max_/Top

# Section C: Synthetic Procedures

## 1. Synthesis of PROTAC-linkers, PROTAC-MMs, and PROTAC-BPDs

### 1.1. Synthesis of S-BPD

**Synthesis of Coupling agent “S”:** synthesized according to the procedures described in literature ^[43]^:

**Synthesis of S-linker:** ARV825 (18.2 mg, 0.020 mmol, 1.0 equiv) and **1** (11.9 mg, 0.030 mmol, 1.5 equiv) were dissolved in MeCN (1 mL) in a microwave (MW) reactor vial. The reaction was heated to 120 °C in a MW reactor for 6 hours where the reaction was deemed to be complete via ^1^H NMR. The reaction mixture was loaded directly on to a neutral alumina column (0–5% MeOH in MeCN) to obtain S-linker as a bright yellow solid (20.6 mg, 0.016 mmol, 79% yield). Trace ARV825 in the ^1^H NMR spectrum can be assigned to release in the NMR solvent.

MALDI-HRMS – Calculated for C_60_H_65_ClN_11_O_12_S^+^: 1198.4218. Found: 1198.429.

^1^H NMR (500 MHz, Acetonitrile-*d*_3_) δ 9.06 (s, 1H), 9.02 – 8.94 (m, 1H), 7.54 – 7.41 (m, 8H), 7.14 (d, *J* = 1.9 Hz, 1H), 7.02 (dd, *J* = 8.9, 6.7 Hz, 3H), 6.96 (dd, *J* = 8.1, 1.3 Hz, 1H), 6.86 – 6.83 (m, 2H), 6.47 (t, *J* = 5.5 Hz, 1H), 5.46 (s, 2H), 4.91 (dd, *J* = 12.0, 5.4 Hz, 1H), 4.82 (t, *J* = 7.1 Hz, 1H), 4.07 – 4.00 (m, 2H), 3.77 – 3.75 (m, 5H), 3.72 – 3.68 (m, 2H), 3.60 (m, 10H), 3.50 – 3.44 (m, 4H), 3.33 (t, *J* = 6.8 Hz, 2H), 2.96 (s, 3H), 2.78 – 2.65 (m, 4H), 2.56 (t, *J* = 7.3 Hz, 2H), 2.48 (s, 3H), 2.15 – 2.05 (m, 2H), 1.76 – 1.57 (m, 9H), 1.49 – 1.41 (m, 2H).

^13^C NMR (126 MHz, Acetonitrile-*d*_3_) δ 172.93, 172.36, 170.48, 170.39, 168.57, 168.29, 165.85, 156.20, 155.70, 152.52, 152.39, 147.82, 141.41, 137.63, 137.08, 137.06, 136.42, 134.48, 133.45, 132.96, 132.39, 131.63, 131.21, 129.61, 128.74, 124.16, 122.48, 122.27, 118.26, 115.52, 114.60, 111.72, 110.82, 71.31, 71.29, 71.21, 71.16, 71.13, 70.25, 70.03, 68.61, 56.75, 55.23, 54.16, 51.93, 49.83, 42.99, 38.76, 34.17, 31.98, 29.08, 26.70, 25.14, 23.24, 14.50, 13.43, 12.09

S-linker: ^1^H NMR assignment

S-linker: ^13^C NMR assignment

**Synthesis of S-MM:** S-linker (14.7 mg, 0.011 mmol, 1.11 equiv) and yne-MM (33.9 mg, 0.01 mmol, 1 equiv) were dissolved in DCM (0.2 mL) under an inert atmosphere. CuOAc (a pinch) was added to the reaction and stirred for 1 h under an inert atmosphere, where the reaction was deemed to be complete when MALDI showed complete consumption of yne-MM. The reaction was then purified using flash column chromatography (neutral alumina, 2–5% MeOH in MeCN) to obtain S-MM as a yellow solid (41.2 mg, 8.7 µmol, 87% yield)

**Synthesis of S-BPD:** In a N_2_-filled glovebox, a 2 mL vial charged with a stir bar containing S-MM (19.1 mg, 4.4 µmol) was added anhydrous THF (66 µL) to completely dissolve the macromonomer. Then Grubbs III catalyst (G3) in THF (0.32 mg, 20 µL, 0.44 µmol) was added quickly all at once to achieve a MM:G3 ratio of 10:1. The reaction was stirred in room temperature for 1 h and the reaction was quenched with the addition of ethyl vinyl ether (50 µL, large excess) and stirred for 5 min. A 1 µL aliquot was taken out for dynamic light scattering analysis, after which the BPD was purified by precipitation in diethyl ether (x3) to give S-BPD (16.3 mg, 82% yield).

**MALDI-HRMS** – Calculated for C_217_H_366_ClN_14_O_84_S^+^: 4591.4202. Found: 4591.210.

### 1.2. Synthesis of M-BPD

**Synthesis of coupling agent “M”: M** was synthesized according to the procedures described in literature ^[43]^:

**Synthesis of M-linker:** ARV825 (14.3 mg, 0.015 mmol, 1.0 equiv) and **2** (11.6 mg, 0.030 mmol, 2 equiv) were dissolved in MeCN (1 mL) in a MW reactor vial. The reaction was heated to 100 °C in a MW reactor for 4.5 hours where reaction was deemed to be complete via ^1^H NMR. The reaction was loaded directly on to a neutral alumina column (0–5% MeOH in MeCN) to obtain M-linker as a bright yellow solid (20.6 mg, 0.018 mmol, 89% yield). Trace impurities in ^1^H NMR can be assigned to ARV825, likely due to release in NMR solvent.

MALDI-HRMS – Calculated for C_59_H_63_ClN_11_O_11_S^+^ : 1168.4113 Found: 1168.467

^1^H NMR (600 MHz, Acetonitrile-*d*_3_) δ 8.92 (s, 1H), 8.79 (s, 1H), 7.53 (t, *J* = 8.7, 7.1 Hz, 1H), 7.50 – 7.47 (m, 4H), 7.46 – 7.41 (m, 4H), 7.05 – 7.00 (m, 4H), 6.86 (d, *J* = 9.0 Hz, 2H), 6.50 – 6.42 (m, 1H), 5.47 (s, 2H), 4.91 (dd, *J* = 12.1, 5.8 Hz, 1H), 4.80 (t, *J* = 7.1 Hz, 1H), 4.07 – 4.03 (m, 2H), 3.75 – 3.73 (m, 2H), 3.68 – 3.65 (m, 2H), 3.62 – 3.56 (m, 9H), 3.48 – 3.42 (m, 4H), 3.31 (t, *J* = 6.7 Hz, 2H), 2.93 (s, 3H), 2.71 – 2.62 (m, 3H), 2.57 – 2.53 (m, 2H), 2.46 (s, 3H), 2.10 – 2.07 (m, 2H), 1.71 – 1.67 (m, 5H), 1.64 – 1.58 (m, 2H), 1.47 – 1.42 (m, 2H).

^13^C NMR (151 MHz, Acetonitrile-*d*_3_) δ 172.97, 172.96, 170.52, 170.45, 168.62, 168.28, 165.95, 156.33, 155.70, 152.49, 147.88, 137.70, 137.11, 136.47, 134.52, 133.50, 132.91, 132.47, 131.54, 131.27, 130.39, 129.66, 128.79, 123.34, 122.35, 118.30, 115.63, 111.78, 110.88, 71.37, 71.26, 71.22, 71.19, 70.31, 70.09, 68.69, 54.98, 54.23, 51.94, 49.88, 43.05, 38.88, 34.52, 32.03, 29.13, 26.78, 25.00, 23.30, 14.56, 13.49, 12.15.

M-linker: ^1^H NMR assignment

M-linker: ^13^C NMR assignment

**Synthesis of M-MM:** M-linker (19.5 mg, 0.015 mmol, 1 equiv) and yne-MM (48.4 mg, 0.014 mmol, 0.95 equiv) were dissolved in DCM (0.2 mL) under an inert atmosphere. CuOAc (a pinch) was added to the reaction and stirred for 1 h under an inert atmosphere, where the reaction was deemed to be complete when MALDI-MS showed complete consumption of yne-MM. The reaction was then purified using flash column chromatography (neutral alumina, 2–10% MeOH in MeCN) to obtain M-MM as a yellow solid (49.6 mg, 10.5 µmol, 73% yield)

**Synthesis of M-BPD:** In a N_2_-filled glovebox, a 250µL vial insert charged with a stir bar containing M-MM (6.1 mg, 1.32 µmol) was added anhydrous THF (20 µL) to completely dissolve the macromonomer. Then Grubbs III catalyst (G3) in THF (0.096 mg, 8 µL, 0.132 µmol) was added quickly all at once to achieve a MM:G3 ratio of 10:1. The reaction was stirred in room temperature for 1 h and the reaction quenched with the addition of ethyl vinyl ether (50 µL, large excess) and stirred for 5 min. A 1µL aliquot was taken out for dynamic light scattering analysis, after which the BPD was purified by precipitation in diethyl ether (x3) to give M-BPD (4.0 mg, 67% yield).

**MALDI-HRMS** – Calculated for C_217_H_364_ClN_14_O_83_S^+^: 4561.4096. Found: 4561.413

### 1.3. Synthesis of F-BPD

**Synthesis of coupling agent “F”: F** was synthesized according to the procedures described in literature ^[43]^:

**Synthesis of F-linker:** ARV825 (15.4 mg, 0.017 mmol, 1.0 equiv) and **3** (12.7 mg, 0.025 mmol, 1.5 equiv) were dissolved in MeCN (1 mL) in a MW reactor vial. The reaction was heated to 90 °C in a MW reactor for 4 hours where reaction was deemed to be complete via ^1^H NMR. The reaction was loaded directly on to a neutral alumina column (0–5% MeOH in MeCN) to obtain F-linker as a bright yellow solid (14.7 mg, 0.011 mmol, 68% yield). Trace impurities in ^1^H NMR can be assigned to ARV825, likely due to release in NMR solvent.

MALDI-HRMS – Calculated for C_64_H_73_ClN_11_O_15_S^+^ : 1302.4692 Found: 1302.322

^1^H NMR (600 MHz, Acetonitrile-*d*_3_) δ 8.91 (s, 1H), 8.63 (s, 1H), 7.53 (dd, *J* = 8.6, 7.1 Hz, 1H), 7.50 – 7.46 (m, 4H), 7.45 – 7.40 (m, 4H), 7.07 – 7.00 (m, 4H), 6.88 – 6.85 (m, 2H), 6.47 (s, 1H), 5.47 (s, 2H), 4.91 (dd, *J* = 12.3, 5.9 Hz, 1H), 4.79 (t, *J* = 7.1 Hz, 1H), 4.07 – 4.02 (m, 2H), 3.79 – 3.76 (m, 2H), 3.76 – 3.73 (m, 2H), 3.68 – 3.65 (m, 2H), 3.62 – 3.53 (m, 22H), 3.47 – 3.41 (m, 4H), 3.36 – 3.33 (m, 2H), 2.92 (s, 3H), 2.78 (t, *J* = 6.1 Hz, 2H), 2.74 – 2.63 (m, 2H), 2.46 (s, 3H), 2.09 – 2.04 (m, 2H), 1.70 (s, 3H).

^13^C NMR (151 MHz, Acetonitrile-*d*_3_) δ 172.95, 171.29, 170.51, 170.43, 168.60, 168.25, 165.91, 156.30, 155.67, 152.42, 147.85, 137.67, 137.10, 136.45, 134.49, 133.48, 132.88, 132.44, 131.57, 131.25, 130.86, 130.45, 129.64, 128.72, 123.32, 122.31, 115.60, 111.76, 110.85, 71.34, 71.24, 71.19, 71.17, 71.13, 71.06, 70.47, 70.29, 70.06, 68.66, 54.95, 54.19, 51.47, 49.86, 43.02, 38.84, 35.93, 32.02, 23.27, 14.55, 13.47, 12.11.

**Synthesis of F-MM:** F-linker (11.5 mg, 8.0 µmol, 1 equiv) and yne-MM (25.8 mg, 7.6 µmol, 0.95 equiv) were dissolved in DCM (0.2 mL) under an inert atmosphere. CuOAc (a pinch) was added to the reaction and stirred for 1 h under an inert atmosphere, where the reaction was deemed to be complete when MALDI-MS showed complete consumption of yne-MM. The reaction was then purified using flash column chromatography (neutral alumina, 100% DCM, then 2–5% MeOH in MeCN) to obtain F-MM as a yellow solid (23.3 mg, 4.95 µmol, 62% yield)

**Synthesis of F-BPD:** In a N_2_-filled glovebox, a 2 mL vial charged with a stir bar containing F-MM (10.84 mg, 2.30 µmol) was added anhydrous THF (35 µL) to completely dissolve the macromonomer. Then Grubbs III catalyst (G3) in THF (0.167 mg, 12 µL, 0.230 µmol) was added quickly all at once to achieve a MM:G3 ratio of 10:1. The reaction was stirred in room temperature for 1 h and the reaction quenched with the addition of ethyl vinyl ether (50 µL, large excess) and stirred for 5 min. A 1µL aliquot was taken out for dynamic light scattering analysis, after which the BPD was purified by precipitation in diethyl ether (x3) to give F-BPD (8.02 mg, 74.3% yield).

**MALDI-HRMS** – Calculated for C_222_H_374_ClN_14_O_87_S^+^ : 4695.4832. Found: 4695.988.

### 1.4. Synthesis of CDK9d-BPD

**Synthesis of CDK9d-linker:** KI-CDK9d-32 (19.9 mg, 0.028 mmol, 1.0 equiv) and **1** (22.7 mg, 0.056 mmol, 2 equiv) were dissolved in anhydrous DMF (0.5 mL) in a MW reactor vial. The reaction was heated to 80 °C in a MW reactor for 3 hours. The reaction was filtered through a 0.22 µm nylon filter and loaded directly on HPLC for separation (0–70% ACN in water) to obtain CDK9d-linker beige solid (10.9 mg, 0.011 mmol, 40% yield).

MALDI-HRMS – Calculated for C_53_H_63_N_12_O_7_^+^ : 979.4938 Found: 979.494

^1^H NMR (600 MHz, DMSO-d6) δ 10.97 – 10.91 (m, 1H), 8.54 (d, J = 7.3 Hz, 1H), 8.05 – 8.00 (m, 3H), 7.74 (d, J = 8.0 Hz, 2H), 7.68 (d, J = 7.7 Hz, 1H), 7.60 (d, J = 8.5 Hz, 1H), 7.34 (d, J = 1.8 Hz, 1H), 7.26 (d, J = 8.1 Hz, 1H), 7.22 (d, J = 8.1 Hz, 1H), 7.17 (s, 1H), 7.13 (d, J = 8.5 Hz, 1H), 6.30 (d, J = 2.2 Hz, 1H), 6.03 (s, 1H), 5.06 (dd, J = 13.3, 5.0 Hz, 1H), 4.91 (s, 2H), 4.74 (s, 2H), 4.54 – 4.49 (m, 1H), 4.35 (d, J = 17.0 Hz, 1H), 4.30 – 4.25 (m, 1H), 4.23 (d, J = 17.0 Hz, 1H), 3.91 – 3.80 (m, 7H), 3.53 – 3.45 (m, 4H), 3.35 (t, J = 6.8 Hz, 2H), 2.99 – 2.83 (m, 2H), 2.61 – 2.58 (m, 4H), 2.38 – 2.33 (m, 1H), 2.28 – 2.21 (m, 1H), 2.20 – 2.10 (m, 2H), 2.06 (dt, J = 13.3, 7.1 Hz, 1H), 1.99 – 1.93 (m, 1H), 1.83 – 1.76 (m, 1H), 1.75 – 1.69 (m, 2H), 1.68 – 1.61 (m, 3H), 1.57 (q, J = 7.3 Hz, 2H), 1.46 – 1.39 (m, 2H), 0.92 (t, J = 7.4 Hz, 3H).

**Synthesis of CDK9d-MM:** CDK9d-linker (10.0 mg, 9.0 µmol, 1 equiv) and yne-MM (27.5 mg, 8.1 µmol, 0.9 equiv) were dissolved in DCM (0.1 mL) and DMF (0.1 mL) an inert atmosphere. CuOAc (a pinch) was added to the reaction and stirred for 1 h under an inert atmosphere, where the reaction was deemed to be complete when MALDI-MS showed complete consumption of yne-MM. The reaction was then purified using flash column chromatography (neutral alumina, 100% DCM, then 20% DMF in MeCN) to obtain CDK9d-linker as a pale-brown solid (13.5 mg, 2.98 µmol, 37% yield)

**Synthesis of CDK9d-BPD:** In a N_2_-filled glovebox, a 2 mL vial charged with a stir bar containing CDK9d-MM (11.1 mg, 2.46 µmol) was added anhydrous DMF (40 µL) to completely dissolve the macromonomer. Then Grubbs III catalyst (G3) in DMF (0.18 mg, 0.25 µmol) was added quickly all at once to achieve a MM:G3 ratio of 10:1. The reaction was stirred in room temperature for 1 h and the reaction quenched with the addition of ethyl vinyl ether (50 µL, large excess) and stirred for 5 min. A 1µL aliquot was taken out for dynamic light scattering analysis, after which the BPD was purified by precipitation in diethyl ether (x3) to give CDK9d-BPD (8.8 mg, 79% yield).

**MALDI-HRMS** – Calculated for C_211_H_364_N_15_O_79_^+^: 4386.5078. Found: 4386.259.

### 1.5. Synthesis of H-BPD

**Synthesis of ARV825H-linker:** ARV825H (285 mg, 0.3 mmol, 1 equiv), AzidoPEG3COOH (140 mg, 0.6 mmol, 2 equiv), Triethylamine (90.9 mg, 0.9 mmol, 3 equiv) and DMAP (3.7 mg, 0.03 mmol) were dissolved in DCM (3 mL). HATU (228 mg, 0.6 mmol, 2 equiv) was then added to the reaction and stirred overnight at r.t, where LCMS showed complete consumption of starting material. The crude was washed with water (3 x 30 mL), washed with brine, dried (Na_2_SO_4_), and concentrated in vacuo. The crude was purified with flash column chromatography (1–6% MeOH in DCM) to obtain ARV825H-linker as a yellow solid (310 mg, 0.266 mmol, 88% yield)

^1^H NMR (500 MHz, Chloroform-*d*) δ 8.83 – 8.74 (m, 1H), 7.51 – 7.42 (m, 3H), 7.41 (dd, *J* = 8.5, 1.9 Hz, 2H), 7.33 (d, *J* = 8.0 Hz, 2H), 7.08 (d, *J* = 7.4 Hz, 1H), 6.89 (dd, *J* = 8.5, 2.2 Hz, 1H), 6.85 – 6.79 (m, 2H), 6.50 – 6.40 (m, 1H), 4.93 – 4.84 (m, 1H), 4.66 (ddd, *J* = 7.9, 6.0, 1.8 Hz, 1H), 4.38 – 4.30 (m, 1H), 4.25 – 4.12 (m, 3H), 4.11 – 4.03 (m, 2H), 3.84 – 3.78 (m, 2H), 3.75 – 3.62 (m, 17H), 3.58 (t, *J* = 4.8 Hz, 2H), 3.49 (ddd, *J* = 14.1, 6.0, 3.1 Hz, 1H), 3.45 – 3.35 (m, 4H), 2.90 – 2.72 (m, 2H), 2.68 (s, 3H), 2.40 (s, 3H), 1.67 (s, 3H).

^13^C NMR (126 MHz, Chloroform-*d*) δ 171.24, 171.22, 170.30, 170.25, 169.31, 168.72, 168.54, 168.53, 167.64, 164.28, 164.24, 155.58, 155.37, 150.06, 146.83, 136.99, 136.41, 136.06, 132.51, 131.65, 131.09, 131.00, 130.48, 130.46, 129.94, 129.93, 128.79, 121.54, 116.82, 114.85, 114.83, 111.72, 110.36, 77.29, 77.04, 76.78, 71.11, 71.08, 70.95, 70.88, 70.69, 70.65, 70.63, 70.60, 70.52, 70.46, 70.06, 70.01, 69.82, 69.78, 68.46, 67.77, 64.00, 63.88, 54.62, 50.69, 50.67, 48.97, 48.94, 42.32, 42.29, 40.32, 31.45, 29.71, 22.80, 14.42, 14.14, 13.13, 11.84.

MALDI-HRMS – Calculated for C_56_H_65_ClN_11_O_14_S^+^ (M+H)^+^: 1182.4116 Found: 1182.477

**Synthesis of H-MM:** ARV825H-linker (58.4 mg, 0.050 mmol, 1.1 equiv) and yne-MM (153 mg, 0.045 mmol, 1 equiv) were dissolved in DCM (0.5 mL) under an inert atmosphere. CuOAc (a pinch) was added to the reaction and stirred for 2 h under an inert atmosphere, where the reaction was deemed to be complete when MALDI showed complete consumption of yne-MM. The reaction was then passed through a neutral alumina plug (flushed with 10% MeOH in Acetone) and concentrated in vacuo. The crude was then purified using recycling preparative HPLC to obtain H-MM as a yellow powder (208 mg, 0.046 mmol, 91% yield)

**Synthesis of H-BPD:** In a N_2_-filled glovebox, a 2 mL charged with a stir bar containing H-MM (36.9 mg, 8.12 µmol) was added anhydrous THF (115 µL) to completely dissolve the macromonomer. Then Grubbs III catalyst (G3) in THF (0.59 mg, 20 µL, 0.812 µmol) was added quickly all at once to achieve a MM:G3 ratio of 10:1. The reaction was stirred in room temperature for 1 h and the reaction quenched with the addition of ethyl vinyl ether (100 µL, large excess) and stirred for 5 min. A 10 µL aliquot was taken out for dynamic light scattering analysis, after which the brush solution was diluted with milliQ water and transferred into a 12kD MWCO dialysis tubing. The solution was dialyzed against milliQ water (2L × 3, water replaced with fresh milliQ water every 8h). The solution in the tubing was then filtered through a 0.22 μm PES filter, then flash frozen using liquid nitrogen and lyophilized to afford H-BPD (30.8 mg, 83% yield)

**MALDI-HRMS** – Calculated for C_215_H_368_ClN_14_O_86_S^+^ (M+H)^+^: 4589.4262, observed 4589.215.

### 1.6. Synthesis of Cy7-S-BPD

**Synthesis of Cy7-MM:** Cy7-azide (5.3 mg, 7.90 µmol, 1.1 equiv) and yne-MM (24.4 mg, 7.18 µmol, 1 equiv) were dissolved in DCM (0.2 mL) under an inert atmosphere. CuOAc (a pinch) was added to the reaction and stirred for 1 h under an inert atmosphere, where the reaction was deemed to be complete when MALDI-MS showed complete consumption of yne-MM. The reaction was then filtered through a neutral alumina plug and then purified using preparatory HPLC (40-45% MeCN in H_2_O) to obtain Cy7-MM (19.2 mg, 4.73 µmol, 66% yield)

**Synthesis of Cy7- S-BPD:** In a N_2_-filled glovebox, a 2 mL vial charged with a stir bar containing S-MM (36.4 mg, 8.00 µmol, 0.95 equiv) and Cy7-MM (1.7 mg, 0.42 µmol, 0.05 equiv) was added anhydrous THF (180 µL) to completely dissolve the macromonomer. Then Grubbs III catalyst (G3) in THF (0.61 mg, 20 µL, 0.842 µmol) was added quickly all at once to achieve a ARV825-S-MM:Cy7-MM:G3 ratio of 9.5:0.5:1. The reaction was stirred in room temperature for 1 h and the reaction quenched with the addition of ethyl vinyl ether (50 µL, large excess) and stirred for 5 min. A 10 µL aliquot was taken out for dynamic light scattering analysis, after which the BPD was purified by precipitation in diethyl ether (x3) to give Cy7-S-BPD (31.7 mg, 83% yield).

**MALDI-HRMS** – Calculated for C_198_H_352_N_9_O_73_^+^ (M-Cl)^+^: 4026.9835, observed 4026.902

## 2. Synthesis of ARV825H

**Full synthetic route of ARV825H**

**Synthesis of 2-(oxiran-2-ylmethoxy)tetrahydro-2H-pyran (1):** Glycidol (14.8 g, 200 mmol), 3,4-Dihydropyran (25.2 g, 300 mmol, 1.5 equiv)) were first dissolved in DCM (300 mL) and then TsOH (344 mg, 0.01 equiv) added to the reaction. The reaction was washed with water (3 x 100mL), then with brine, dried (Na_2_SO_4_) and concentrated in vacuo. The crude was purified with flash column chromatography (25% Et_2_O in Hexanes) to obtain a clear oil (19.4 g, 12.3 mmol, 61% yield).

**Synthesis of 2-azidoethanol:** 2 bromoethanol (18.7 g, 150 mmol) and NaN_3_ (14.6 g, 225 mmol) were dissolved in water (100 mL) and heated to 80 °C fitted with a reflux condenser. The reaction was stirred overnight an extracted with Et_2_O (3 x 50 mL). The combined organic fractions were washed with brine, dried (MgSO_4_) and concentrated in vacuo at room temperature to give 2-azidoethanol (11.1 g, 127 mmol, 85% yield) as a clear liquid. As a safety measure, the product was stored as an 60% w.t. solution in benzene at -20 °C.

**Synthesis of 1-(2-azidoethoxy)-3-((tetrahydro-2H-pyran-2-yl)oxy)propan-2-ol (2):** A flame-dried RBF with a magnetic stir bar was charged with 2-azidoethanol in benzene (7.25 g, 50 mmol, 2.0 equiv) and dissolved in dry DMF (50 mL). The flask was cooled to 0 °C and NaH (24 g, 60 mmol, 3 equiv) was added in 3 batches to the reaction. When the bubbling has ceased and the reaction returned to room temperature, epoxide **1** (3.16 g, 20 mmol, 1.0 equiv) was then added dropwise into the reaction and stirred at r.t under an inert atmosphere for 24 h. The reaction was quenched with 1 M NH_4_Cl and then extracted with EtOAc (3 x 50 mL). The combined fractions were washed with brine, dried (MgSO_4_), and concentrated in vacuo. The crude was then purified with flash column chromatography (15–25% EtOAc in Hexanes) to afford **3** as a faint-yellow oil (2.92 g, 11.8 mmol, 59% yield).

^1^H NMR (400 MHz, Chloroform-*d*) δ 4.56 (m, 1H), 4.01 – 3.86 (m, 2H), 3.82 – 3.49 (m, 7H), 3.39 (t, *J* = 5.0 Hz, 2H), 3.10 – 2.19 (br s, 1H), 1.90 – 1.71 (m, 2H), 1.64 – 1.48 (m, 4H).

^13^C NMR (100 MHz, Chloroform-*d*) δ 100.42, 100.34, 72.54, 72.48, 70.77, 70.56, 70.49, 69.87, 69.84, 69.65, 63.39, 63.34, 50.85, 30.86, 30.83, 25.36, 20.15, 20.07.

DART HRMS – Calculated for C_11_H_19_N_3_O_4_^+^ (M+H)^+^: 246.1148. Found: 246.1196

**Synthesis of 2-(2-((1-(2-azidoethoxy)-3-((tetrahydro-2H-pyran-2-yl)oxy)propan-2-yl)oxy)ethoxy) ethyl4-methylbenzenesulfonate (3):** A flame-dried RBF with a magnetic stir bar was charged with **2** (1.47 g, 6 mmol, 1.0 equiv) and dissolved in dry DMF (50 mL). The setup was cooled to 0 °C and NaH (288 mg, 12.0 mmol, 1.2 equiv) was added and stirred for 1 h. When the reaction was no longer bubbling, TsO-PEG2-OTs (9.95 g, 24.0 mmol, 4 equiv) was added to the reaction and stirred overnight at r.t. The reaction was quenched with 1M NH_4_Cl, and extracted with EtOAc (3 x 70 mL). The combined fractions were washed with water (3 x 100 mL), then washed with brine, dried (MgSO4), and concentrated in vacuo. The crude was then dissolved in EtOH to crystallize excess TsO-PEG2-OTs remaining in solution. The filtrate was then concentrated in vacuo and purified with flash column chromatography (30 – 50% EtOAc in Hexanes) to obtain **3** as a light-yellow oil (988 mg, 2.03 mmol, 34% yield)

^1^H NMR (500 MHz, Chloroform-*d*) δ 7.80 (d, *J* = 8.4 Hz, 2H), 7.34 (d, *J* = 8.2 Hz, 2H), 4.60 (dd, *J* = 4.3, 2.9 Hz, 1H), 4.15 (t, *J* = 4.9 Hz, 2H), 3.89 – 3.45 (m, 16H), 3.35 (t, *J* = 4.9 Hz, 2H), 2.45 (s, 3H), 1.80 (m, 1H), 1.74 – 1.65 (m, 1H), 1.62 – 1.47 (m, 1H).

^13^C NMR (126 MHz, Chloroform-*d*) δ 144.80, 133.04, 129.84, 128.00, 99.21, 98.97, 78.61, 78.51, 71.61, 71.54, 70.98, 70.35, 70.03, 69.90, 69.27, 68.67, 67.28, 66.83, 62.27, 62.15, 50.78, 30.59, 30.54, 25.43, 21.66, 19.49, 19.41.

DART-HRMS – Calculated for C_21_H_34_N_3_O_4_S^+^ (M+H)^+^: 488.2061. Found: 488.2001

**Synthesis of** **(4):** To a 10 mL microwave vial was added **3** (1.17 g, 2.4 mmol, 1.5 equiv), OTX015 (787 mg, 1.6 mmol, 1 equiv), K_2_CO_3_ (288 mg, 2.08 mmol, 1.3 equiv), and finally MeCN (5 mL). The reaction was capped and heated to 90 °C in a microwave reactor and stirred for 20 hours. The reaction was first concentrated to dryness and then resuspended in DCM (30 mL). The solution was washed with water (5 x 50 mL), washed with brine, dried (Na_2_SO_4_), and concentrated in vacuo. The crude was purified with flash column chromatography (1 – 3% MeOH in DCM) to obtain **4** as a brown solid (915 mg, 1.13 mmol, 71% yield)

^1^H NMR (500 MHz, Chloroform-*d*) δ 8.73 (s, 1H), 7.45 (d, *J* = 9.0 Hz, 2H), 7.40 (d, *J* = 8.3 Hz, 2H), 7.31 (d, *J* = 8.3 Hz, 2H), 6.84 (d, *J* = 9.0 Hz, 2H), 4.64 (dd, *J* = 8.1, 5.9 Hz, 1H), 4.60 (t, *J* = 3.6 Hz, 1H), 4.07 (t, *J* = 4.9 Hz, 2H), 3.86 – 3.46 (m, 21H), 3.35 – 3.32 (m, 2H), 2.66 (s, 3H), 2.39 (s, 3H), 1.84 – 1.75 (m, 2H), 1.66 (s, 3H), 1.61 – 1.45 (m, 6H).

^13^C NMR (126 MHz, Chloroform-*d*) δ 168.67, 164.17, 155.84, 155.58, 150.10, 136.99, 136.64, 132.23, 131.65, 131.07, 131.05, 130.55, 129.98, 128.86, 121.61, 114.96, 99.28, 99.02, 78.70, 78.59, 77.41, 77.16, 76.91, 71.69, 71.62, 71.15, 70.44, 70.19, 70.07, 69.83, 67.85, 67.41, 66.93, 62.31, 62.18, 54.77, 50.87, 40.63, 31.55, 30.69, 30.63, 25.54, 19.55, 19.47, 14.52, 13.22, 11.96.

QTof-HRMS – Calculated for C_39_H_48_ClN_8_O_7_S^+^ (M+H)^+^: 807.3050. Found: 807.3091

**Synthesis of** **(5):** Compound **4** (888 mg, 1.1 mmol, 1.0 equiv) and PPh_3_ (346 mg, 1.32 mmol, 1.2 equiv) were dissolved in MeCN (5 mL). To the reaction was added 2 drops of water and stirred at room temperature for 3 h. The reaction was then evaporated to dryness and redissolved in DCM. The organic layer was washed with water (3 x 30 mL), washed with brine, dried (Na_2_SO_4_), and concentrated in vacuo. The crude was then taken to the next step without purification.

Crude material from previous step (~859 mg) was added to a 10mL microwave vial and dissolved in MeCN (5 mL). To the reaction was then added 4-fluorothalidomide (911 mg, 3.30 mmol, 3 equiv) and DIPEA (427 mg, 3.30 mmol, 3 equiv). The reaction was capped and heated to 95°C for 12 hr, where LCMS showed complete consumption of the intermediate. The reaction was then evaporated to dryness then redissolved in DCM. The organic layer was washed with water (3 x 30 mL), washed with brine, dried (Na_2_SO_4_), and concentrated in vacuo. The crude was purified with flash column chromatography (5% MeOH in DCM) to obtain **5** as a bright-yellow solid (607 mg, 0.585 mmol, 53% yield over 2 steps)

^1^H NMR (600 MHz, DMSO-d6) δ 11.08 (s, 1H), 10.15 (s, 1H), 7.56 (dd, J = 8.6, 7.0 Hz, 1H), 7.54 – 7.50 (m, 2H), 7.48 (d, J = 8.5 Hz, 2H), 7.44 – 7.38 (m, 2H), 7.12 (d, J = 8.6 Hz, 1H), 7.03 (d, J = 7.0 Hz, 1H), 6.90 – 6.84 (m, 2H), 6.60 (t, J = 5.9 Hz, 1H), 5.05 (dd, J = 12.8, 5.4 Hz, 1H), 4.59 (t, J = 7.1 Hz, 1H), 4.52 (m, 1H), 4.02 (t, J = 4.6 Hz, 2H), 3.70 (m, 2H), 3.67 – 3.64 (m, 2H), 3.63 – 3.60 (m, 2H), 3.48 – 3.33 (m, 27H), 2.87 (ddd, J = 16.8, 13.7, 5.4 Hz, 1H), 2.60 (s, 3H), 2.42 (s, 3H), 2.01 (m, 1H), 1.63 (s, 3H), 1.45 – 1.35 – 1.47 (m, 4H).

^13^C NMR (151 MHz, DMSO) δ 173.24, 170.50, 169.41, 168.53, 167.75, 163.65, 155.54, 154.79, 150.36, 146.92, 137.20, 136.63, 135.71, 132.96, 132.77, 132.53, 131.22, 130.60, 130.33, 130.03, 128.97, 121.00, 117.97, 114.92, 111.12, 109.69, 98.74, 98.38, 78.03, 70.82, 70.68, 70.66, 69.79, 69.53, 69.43, 67.73, 61.65, 61.54, 61.50, 54.27, 49.02, 42.14, 38.95, 34.66, 31.43, 25.46, 25.25, 22.53, 14.44, 13.16, 11.78.

QToF HRMS – Calculated for C_52_H_58_ClN_8_O_11_S^+^ (M+H)^+^: 1037.3629. Found: 1037.3583

**Synthesis of** **ARV825H:** Compound **5** (607 mg, 0.59 mmol) was added to a 8:1:1 mixture of DCM/H_2_O/TFA (5 mL) and stirred at room temperature for 1 hour. Upon complete deprotection, DCM (40 mL) was added to the reaction. The reaction was washed with Na_2_CO_3_, then with brine, dried (Na_2_SO_4_) and concentrated in vacuo. The crude was purified using flash column chromatography (6% MeOH in DCM) to obtain **ARV825H** (520 mg, 0.54 mmol, 92% yield) as a bright yellow solid.

^1^H NMR (500 MHz, DMSO-*d*_6_) δ 11.09 (s, 1H), 10.16 (s, 1H), 7.56 (dd, *J* = 8.5, 7.1 Hz, 1H), 7.52 (d, *J* = 8.7 Hz, 2H), 7.48 (d, *J* = 8.7 Hz, 2H), 7.42 (d, *J* = 8.6 Hz, 2H), 7.12 (d, *J* = 8.6 Hz, 1H), 7.03 (d, *J* = 7.0 Hz, 1H), 6.88 (d, *J* = 8.6 Hz, 2H), 6.60 (t, *J* = 5.8 Hz, 1H), 5.05 (dd, *J* = 12.9, 5.4 Hz, 1H), 4.61 – 4.54 (m, 2H), 4.01 (dd, *J* = 5.7, 3.7 Hz, 2H), 3.70 (t, *J* = 4.7 Hz, 2H), 3.67 – 3.63 (m, 2H), 3.61 (t, *J* = 5.4 Hz, 2H), 3.57 – 3.39 (m, 12H), 2.87 (ddd, J = 16.8, 13.7, 5.4 Hz, 1H), 2.60 (s, 3H), 2.42 (s, 3H), 2.01 (m, 1H), 1.63 (s, 3H).

^13^C NMR (126 MHz, DMSO-*d*_6_) δ 173.27, 170.55, 169.41, 168.55, 167.77, 163.66, 155.55, 154.79, 150.37, 146.91, 137.21, 136.66, 135.72, 132.96, 132.77, 132.54, 131.22, 130.61, 130.34, 130.04, 128.98, 121.01, 117.96, 114.93, 111.13, 109.69, 80.22, 71.19, 70.68, 69.72, 69.43, 69.39, 67.70, 61.20, 54.27, 49.02, 42.19, 40.50, 40.33, 40.16, 40.00, 39.83, 39.66, 39.50, 38.96, 31.45, 22.61, 14.54, 13.17, 11.79.

QTof-HRMS Calculated for C_48_H_49_ClN_8_O_10_S^+^ (M+H)^+^: 953.3054. Found: 953.3096

# NMR Spectra

**ARV825-S-linker**

^1^H NMR

^13^C NMR

^1^H-^1^H COSY

^1^H-^13^C HMBC

**ARV825-M-linker**

^1^H NMR

^13^C NMR

^1^H-^13^C HMBC

**ARV825-F-linker**

^1^H NMR

**CDK9d-linker**

^1^H NMR

^13^C NMR

^1^H COSY

^1^H NOESY

^1^H-^13^C HMBC

**ARV825H-linker**

^1^H NMR

^13^C NMR

1-(2-azidoethoxy)-3-((tetrahydro-2H-pyran-2-yl)oxy)propan-2-ol **(2)**

^1^H NMR

^13^C NMR

2-(2-((1-(2-azidoethoxy)-3-((tetrahydro-2H-pyran-2-yl)oxy)propan-2-yl)oxy)ethoxy)ethyl4-methylbenzenesulfonate **(3)**

^1^H NMR

^13^C NMR

**Compound 4**

^1^H NMR

^13^C NMR

**Compound 5**

^1^H NMR

^13^C NMR

**ARV825H**

^1^H NMR

^13^C NMR

# MALDI-MS Spectra

**ARV825-S-linker: C_60_H_65_ClN_11_O_12_S^+^**

Calcd m/z: 1198.4218 (100.0%), 1199.4252 (64.9%), 1200.4189 (32.0%), 1201.4222 (20.8%), 1200.4286 (20.7%), 1202.4256 (6.6%), 1200.4176 (4.5%)

Found: 1198.429

**ARV825-M-linker: C_59_H_63_ClN_11_O_11_S^+^**

Calcd m/z: 1168.4113 (100.0%), 1169.4146 (63.8%), 1170.4083 (32.0%), 1171.4117 (20.4%), 1170.4180 (20.0%), 1172.4150 (6.4%), 1170.4071 (4.5%)

Found: 1168.467

**ARV825-F-linker:** **C_64_H_73_ClN_11_O_15_S^+^**

Calcd m/z: 1302.4692 (100.0%), 1303.4725 (69.2%), 1304.4662 (32.0%), 1304.4759 (23.6%), 1305.4696 (22.1%), 1306.4729 (7.5%), 1305.4792 (5.3%), 1304.4650 (4.5%)

Found: 1302.322

**CDK9d-linker: C_53_H_63_N_12_O_7_^+^**

Calcd m/z: 979.4938 (100.0%), 980.4971 (57.3%), 981.5005 (16.1%), 980.4908 (4.4%), 982.5038 (3.0%), 981.4942 (2.5%), 981.4980 (1.4%)

Found: 979.494

**ARV825-S-MM:**

Calculated for C_217_H_366_ClN_14_O_84_S^+^: 4591.4202. Found: 4591.210. Calculated mass corresponds to PEG degree of polymerization = 68. Distribution of peaks spaced at 44 amu is observed due to polydispersity of sample. A minor secondary distribution is observed corresponding to (M-H+Na)^+^.

**ARV825-M-MM**

Calculated for C_217_H_364_ClN_14_O_83_S^+^: 4561.4096. Found: 4561.413

Calculated mass corresponds to PEG degree of polymerization = 68. Distribution of peaks spaced at 44 amu is observed due to polydispersity of sample. A minor secondary distribution is observed corresponding to (M-H+Na)^+^.

**ARV825-F-MM**

Calculated for C_222_H_374_ClN_14_O_87_S^+^ : 4695.4832. Found: 4695.988

Calculated mass corresponds to PEG degree of polymerization = 68. Distribution of peaks spaced at 44 amu is observed due to polydispersity of sample. A minor secondary distribution is observed corresponding to (M-H+Na)^+^.

**CDK9d-MM:**

Calculated for C_211_H_364_N_15_O_79_^+^: 4386.5078. Found: 4386.259

Calculated mass corresponds to PEG degree of polymerization = 68. Distribution of peaks spaced at 44 amu is observed due to polydispersity of sample. A secondary distribution is observed corresponding to (M-H+Na)^+^.

(M-H+Na)^+^ Distribution

**ARV825H-MM:**

Calculated for C_215_H_368_ClN_14_O_86_S^+^ (M+H)^+^: 4589.4262, observed 4589.215.

Calculated mass corresponds to PEG degree of polymerization = 68. Distribution of peaks spaced at 44 amu is observed due to polydispersity of sample. A minor secondary distribution is observed corresponding to (M+Na)^+^.

**Cy7-MM**

Calculated for C_198_H_352_N_9_O_73_^+^ (M-Cl)^+^: 4026.9835, observed 4026.902:
